# Ginseng-containing traditional medicine preparations in combination with fluoropyrimidine-based chemotherapy for advanced gastric cancer: A systematic review and meta-analysis

**DOI:** 10.1371/journal.pone.0284398

**Published:** 2023-04-17

**Authors:** Jiaqi Hu, Mengqi Cheng, Yue Li, Bolun Shi, Shulin He, Ziang Yao, Juling Jiang, Huibo Yu, Zhongning He, Yuwei Zhao, Honggang Zheng, Baojin Hua, Rui Liu

**Affiliations:** 1 Department of Oncology, Guang’anmen Hospital, China Academy of Chinese Medical Sciences, Beijing, China; 2 Graduate School, Beijing University of Chinese Medicine, Beijing, China; 3 The First Affiliated Hospital of Zhejiang Chinese Medical University (Zhejiang Provincial Hospital of Chinese Medicine), Hangzhou, China; 4 Xiyuan Hospital, China Academy of Chinese Medical Sciences, Beijing, China; Jiangsu University, CHINA

## Abstract

**Background:**

Ginseng-containing traditional medicine preparations (G-TMPs) in combination with fluoropyrimidine-based chemotherapy (FBC) are well-known treatments for advanced gastric cancer (AGC), with a superior efficacy to FBC alone. However, evidence regarding their efficacy remains limited. The purpose of this meta-analysis is to evaluate the efficacy and safety of G-TMPs in combination with FBC for the treatment of AGC.

**Methods:**

Eight electronic databases were searched for randomized controlled trials (RCTs) using G-TMPs with FBC for the treatment of AGC. The primary outcome included the tumor response, while the secondary outcomes included the quality of life (QoL), proportions of peripheral blood lymphocytes, adverse drug reactions (ADRs), and levels of cancer biomarkers. The quality of evidence for each outcome was assessed using GRADE profilers.

**Results:**

A total of 1,960 participants were involved in the 26 RCTs included. Patients treated with FBC plus G-TMPs had better objective response (risk ratio [RR] = 1.23, 95% confidence interval [CI]: 1.13 to 1.35, p < 0.00001) and disease control (RR = 1.13, 95% CI: 1.08 to 1.19, p < 0.00001) rates than those treated with FBC alone. Additionally, the combination group had a better QoL, higher proportions of CD3^+^ T cells, CD4^+^ T cells, and natural killer cells, as well as a higher CD4^+^/CD8^+^ T-cell ratio. Furthermore, lower levels of CA19-9, CA72-4, and CEA were confirmed in the combination treatment group. In addition, G-TMPs reduced the incidence of ADRs during chemotherapy.

**Conclusion:**

In combination with FBC, G-TMPs can potentially enhance efficacy, reduce ADRs, and improve prognosis for patients with AGC. However, high-quality randomized studies remain warranted.

**Systematic review registration:**

PROSPERO Number: CRD42021264938.

## Introduction

Gastric cancer is a malignant tumor that imposes a heavy burden on individuals, families, and health care systems. From 2007 to 2017, the incidence of gastric cancer rose by 0.25%, and this disease ranked third highest globally in terms of disability-adjusted life years (19.1 million in 2017) [1]. According to the Global Cancer Statistics, the mortality rate of gastric cancer in 2020 was 7.7%, accounting for 1 in every 13 deaths and ranking fourth among all malignant tumors [2]. Unfortunately, most patients with gastric cancer are diagnosed too late for the surgically removal of cancer tissue [3]. Advanced gastric cancer (AGC) is treated with fluoropyrimidine-based chemotherapy (FBC) worldwide. It is advised that fluoropyrimidines (fluorouracils, capecitabines (CAP), and tegafur) be used in combination with platinum (cisplatin [DDP] or oxaliplatin [OXA]) according to the National Comprehensive Cancer Network guidelines (version 4.2021) [4] and Chinese Society of Clinical Oncology guidelines (version 2021) [5]. The median progression-free survival of patients on FBC is approximately 5 months, while the median overall survival is only approximately 15 months. Moreover, FBC is accompanied by adverse reactions, such as anemia, neutropenia, and diarrhea, as well as by treatment-related deaths from pulmonary tuberculosis and viral pneumonia [6–8], which are urgent problems remain to be addressed.

Traditional medicine preparations (TMPs) have been confirmed to enhance the efficacy of chemotherapy for AGC, including the objective response rate (ORR) and disease control rate (DCR), and improve the quality of life (QoL) of patients. Chemotherapy-induced gastrointestinal reactions, bone marrow suppression, and hand-foot syndrome were also obviously alleviated [9–11]. Ginseng (also called renshen in Chinese), the dry root and rhizome of *Panax ginseng* C.A. Mey. (Araliaceae) is an important component of traditional Chinese preparations owing to its medicinal value, such as enhancing the effect of chemotherapy on malignant tumors and reducing adverse reactions and toxicity [12, 13]. A meta-analysis has shown that ginseng and its ingredients promote chemotherapy effects in patients with non-small cell lung cancer, in particular by enhancing the tumor response, improving immunity, and reducing adverse drug reactions (ADRs) [14]. However, the effect of ginseng-containing TMPs (G-TMPs) on the treatment of patients with AGC remains unknown.

To date, there have been few clinical trials on the use of G-TMPs in combination with FBC for the treatment of AGC, and their results were less convincing owing to small sample sizes. Therefore, this meta-analysis systematically evaluates the efficacy and safety of G-TMPs in combination with FBC for the treatment of AGC.

## Materials and methods

### Study design

This study was conducted according to the PRISMA guidelines [15] and registered in PROSPERO under number CRD42021264938.

### Inclusion criteria

#### Patients

Patients diagnosed with gastric cancer in stages III–IV according to the TNM staging system were included. There were no restrictions on sex or age.

#### Interventions

G-TMPs were administered in combination with FBC to the experimental group. The preparations that included ginseng could be proprietary formulations or herbal concoctions that were self-prepared and recommended by medical professionals. There were no restrictions on the dosage forms, including injections, decoctions, or capsules. The definition of ginseng herein was the dry root and rhizome of *P*. *ginseng*. The control group received FBC alone.

#### Primary outcome

Both the ORR and DCR, which were determined independently before and after the trial, were used to evaluate the tumor response as the primary outcome, according to the World Health Organization (WHO) [16] and RECIST [17] criteria.

#### Secondary outcomes

The QoL, proportions of peripheral blood lymphocytes, and levels of cancer biomarkers were the secondary outcomes. ADRs were also analyzed to evaluate the safety. When the Karnofsky Performance Status (KPS) score decreased after therapy by fewer than 10 points, the therapy was regarded to be helpful for QoL. It was also permissible to perform a comparison between the mean ± standardized difference (SD) values of the KPS scores obtained before and after therapy. The proportions of CD3^+^ and CD4^+^ T lymphocytes and the CD4^+^/CD8^+^ T-lymphocyte ratio were determined in peripheral blood. The percentage of natural killer (NK) cells was also estimated. Before the beginning of each study and at the conclusion of the follow-up period, the levels of the cancer biomarkers CA19-9, CA72-4, and CEA were measured. Synthesizing the mean ± SD changes helped analyze group differences. ADRs were examined by determining the number of patients with stage 0–IV cancer who experienced gastrointestinal toxicity (diarrhea, nausea, and vomiting), hematotoxicity (decreases in hemoglobin levels and platelet and white blood cell [WBC] counts), liver dysfunction, and renal failure, according to the WHO recommendations [16]. When patients experienced level II–IV ADRs, the treatment was considered a contributing factor.

#### Types of studies

We examined every single randomized controlled trial (RCT) published in either English or Chinese; quasi-randomized studies were excluded. Only full journal papers with sufficient data for analysis were included.

### Exclusion criteria

The following exclusion criteria were used: (1) a diagnosis of gastric cardia cancer; (2) a history of radiotherapy, chemotherapy, or any other antitumor treatment within 1 month of the trial, concomitant infection, additional malignant tumors, or other serious diseases; (3) the ginseng-containing preparation was not fixed within the study; (4) unclear or contradictory observation points between the two groups in the same study; (5) inadequate information.

### Search strategy

The Cochrane Library, EMBASE, PubMed, clinicaltrials.gov, Wangfang Data Knowledge Service Platform, China National Knowledge Infrastructure, Chinese Biomedical Literature Database, and Chinese Scientific Journal Database (VIP database) were searched from conception to June 29, 2021. The following terms were used in the database searches conducted in English: "stomach neoplasms," "gastr*," "stomach*," "digest*," "epigastr*," "panax," "ginsenosides," "renshen," "schinseng," "ginsan," "ginseng*," "shinseng," "ninjin," "gingilone," "panaxoside*," "ginsenoside*," "protopanaxa*," "protopanaxadiol," "protopanaxatriol," "panaxagin," "ginsenol," and "ginsenine." For the Chinese databases, comparable keywords were used (the detailed search strategy may be found in S1 File). More trials were sought by examining the reference lists of previous studies that were related to the use of G-TMPs in combination with FBC for the treatment of AGC. Two reviewers (Y. Li and B. Shi) independently screened all the literature. During this process, any disagreements that arose were settled either by reaching a consensus or by a third reviewer (J. Hu).

### Data extraction

The reviews were individually entered into the Endnote X9 program by two different reviewers (Y. Zhao and M. Cheng). After excluding the duplicate studies, two reviewers (J. Hu and M. Cheng) independently evaluated the remaining studies. A third reviewer (H. Zheng) was brought in to discuss and resolve any differences of opinion that arose throughout the course of this process. Data were entered into Excel by S. He and H. Yu. Basic information, methods, and outcomes were extracted from the data. When the necessary data were missing, we explained this in our article.

### Evaluation of the risk of bias

According to the Cochrane Handbook (version 5.1.0) [18], Z. He, J. Jiang, and Z. Yao analyzed the included papers using the Cochrane risk of bias method for randomized trials. By consensus or with the help of another reviewer (H. Zheng), any disagreements that arose throughout this process were resolved.

### Statistical analysis

#### Data synthesis

Using Review Manager 5.3, a meta-analysis was carried out on the included studies by two reviewers, Y. Li and Z. Yao. The dichotomous data were presented using the risk ratio (RR), whereas continuous data were presented using the standardized mean difference (SMD). A p value of < 0.05 indicated statistical significance, and 95% confidence intervals (CIs) were provided. Heterogeneity was assessed using Cochran’s Q test and the *I*^2^ statistic. An *I*^2^ index of > 50% indicated a high statistical heterogeneity among different trials. A fixed-effects model (FEM) was utilized to generate the RR, SMD, and their 95% CI when the heterogeneity was low (p ≥ 0.10, *I*^2^ ≤ 50%). In all other cases, a random-effects model (REM) was applied. To test the robustness of the findings, a sensitivity analysis was conducted by successively eliminating each study. When more than 10 papers were included, publication bias was examined using the nonparametric trim-and-fill test and Egger’s test.

#### Subgroup analysis

Subgroup analysis was undertaken to reveal the clinical heterogeneity and its effect on outcomes based on the KPS score, therapeutic process, drug administration of G-TMPs, chemotherapy regimen, follow-up period, and various combinations of ginseng and other herbs.

### Assessment of evidence quality

Independent analyses were conducted by S. He and M. Cheng to determine the quality of the evidence supporting each outcome using GRADE profilers [19]. Any disagreements were resolved by consensus or by another reviewer (H. Zheng).

## Results

### Literature screening results

**Fig 1:**
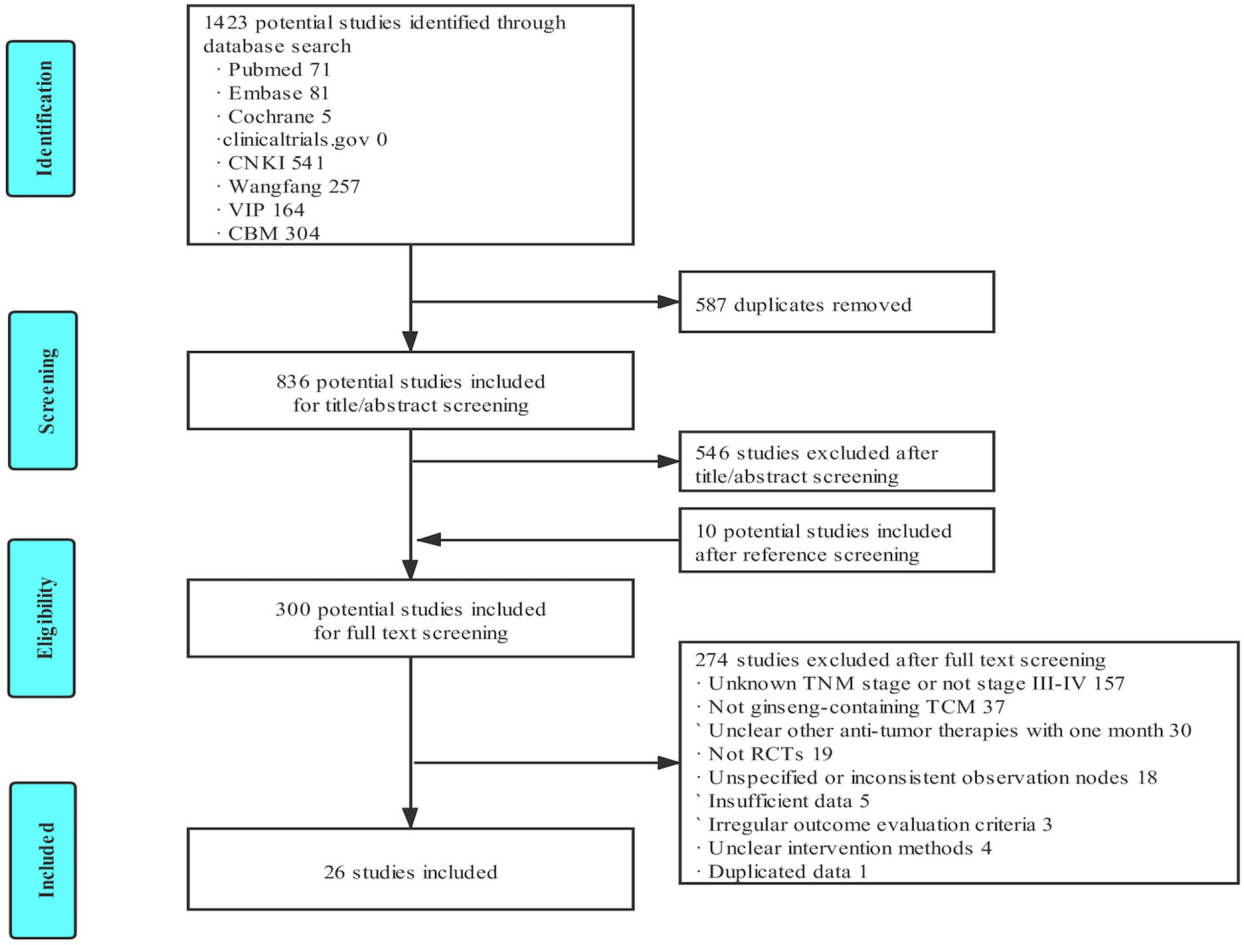
Flow diagram. TCM, traditional Chinese medicine; RCTs, randomized controlled trials.

During the initial search, a total of 1,423 articles were found; after removing 587 duplicate articles, 836 articles remained, of which 290 articles were selected after screening the titles and abstracts. The references in related reviews were also screened, and 10 additional studies were included. In the final meta-analysis, 26 eligible studies were included, which fulfilled the criteria after the complete text was read. Fig 1 shows the process of the literature search.

### Study characteristics

In the 26 RCTs, 1,960 patients with stage III–IV gastric cancer (981 in the experimental group and 979 in the control group) were included. Table 1 depicts the basic characteristics of the included studies. All the studies were carried out in China and published in Chinese journals between 2004 and 2020 [20–45]. None of the included individuals had received antitumor therapy within 1 month before the start of the study. The individuals in 14 trials [20, 22, 24, 26–28, 31–33, 36, 37, 40, 44, 45] received the primary treatment, while this information was unclear in the other trials. Thirteen trials [21–24, 26, 27, 32, 33, 35, 39–41, 45] included participants with KPS scores ≥50 or ≥60; nine trials [25, 28, 29, 31, 36, 42–44] included participants with KPS scores ≥70 or ≥80; and four trials [20, 30, 34, 38] included participants with unclear KPS scores. Regarding the fluoropyrimidines used, 14 trials [20, 22, 23, 27, 30–32, 34, 35, 38, 39, 42–44] used a 5-fluorouracil (5-FU)-based chemotherapy regimen, 8 trials [21, 24, 26, 29, 33, 40, 41, 45] used an S-1-based chemotherapy regimen, and 4 trials [25, 28, 36, 37] used a CAP-based chemotherapy regimen. Regarding the use of platinum, 10 trials [22, 23, 27, 30–32, 34, 35, 39, 44] used DDP-based chemotherapy, and 12 trials [20, 24–26, 28, 37, 38, 40–43, 45] used OXA-based chemotherapy, whereas 4 trials [21, 29, 33, 36] did not use platinum. All studies included a follow-up period of 2 to 18 weeks. Furthermore, 23 studies reported tumor responses using the WHO or RECIST guidelines, 16 trials reported QoL using the KPS, 9 trials reported peripheral blood lymphocyte levels, 5 trials reported the levels of cancer biomarkers before and after treatment, and 11 trials reported ADRs using the WHO chemotherapy toxicity response grading criteria.

**Table 1:** Main characteristics of studies included in the meta-analysis.

First Author and Publication Year	Advanced Gastric Cancer (AGC, III-IV)						Interventions					Follow-up	Outcomes
	KPS Score	TP	E/C	M/F	TNM Stage	Age(E/C), mean or mean ± SD	G-TMPs/Quality Control	Specific Components	Potential active ingredients related to *Ginseng*	Drug Delivery	FBC Regimen		
An et al., 2012	Unclear	PT	38/32	36/34	III, IV	45–70/44–71	Kangai Injection: 60 ml, qd, d1–14, 21 d/C, 2 Cycles/ An approved drug and has a marketing authorization in China	*P*. *ginseng*, *A*. *mongholicus*, and *Sophora flavescens*	*Ginsenosides (Rf*, *Rb2*, *Rc*, *Rd*, and *Rb1)* [46]	Intravenously	OXA+CF+Fu: OXA: 85 mg/m^2^, d1; CF: 200 mg/m^2^, d1–2; Fu: 400 mg/m^2^ first and 600 mg/m^2^ following, d1–2, 21 d/C, 2 Cycles	6 w	O1,2,4,5
Deng and Li, 2018	≥ 60	Unclear	31/31	35/27	IIIb, IV	64.89 ± 10.22/65.31 ± 10.91	Shenmai Injection: 50 ml, qd, d1–7, 21 d/C, 3 Cycles/ An approved drug and has a marketing authorization in China	*P*. *ginseng* and *O*. *japonicus*	Unclear	Intravenously	S-1: body surface area < 1.25 m^2^: 40 mg, bid; 1.25 m^2^ < body surface area < 1.5 m^2^: 50mg, bid; 1.5 m^2^ < body surface area: 60 mg, bid; d1–14, 21 d/C, 3 Cycles	9 w	O1,2,5,6
Duan, 2016	> 60	PT	46/46	51/41	IIIb: 68, IV: 24	51.88 ± 5.91/52.50 ± 6.14	Aidi Injection: 50 ml, qd, d1–21, 21 d/C, 2 Cycles/ An approved drug and has a marketing authorization in China	*P*. *ginseng*, *A*. *mongholicus*, *Mylabris*, and *E*. *senticosus*	*Ginsenosides (Rg1*, *Re*, and *Rb1)* [47]	Intravenously	TAX+DDP+Fu: TAX: 175 mg/m^2^, d1; DDP: 25 mg/m^2^, d1-5; Fu: 600 mg/m^2^, d5, 21 d/C, 2 Cycles	6 w	O1,2
Gong et al., 2006	≥ 60	Unclear	26/30	31/25	IIIb: 36, IV: 20	54.2/55.4	Aidi Injection: 50 ml, qd, d1–21, 21d/C, 4 Cycles/ An approved drug and has a marketing authorization in China	*P*. *ginseng*, *A*. *mongholicus*, *Mylabris*, and *E*. *senticosus*	*Ginsenosides (Rg1*, *Re*, and *Rb1)* [c]	Intravenously	TAX+5-Fu+CF+DDP: TAX: 135 mg/m^2^, d1; 5-Fu: 500 mg/(m^2^·d), 4 h/d, d1–5; CF: 100 mg/(m^2^·d), d1–5; DDP: 30 mg/(m^2^·d), d1–3, 21 d/C, 4 Cycles	12 w	O5
Hou and Yan, 2020	> 70	Unclear	45/45	49/41	IV	66.07 ± 8.54/65.84 ± 8.11	*Ginseng*-containing Decoction: d1–42d/ Unclear	*P*. *ginseng* 6 g, *A*. *mongholicus* 18 g, *A*. *macrocephala* 9 g, *G*. *uralensis* 9 g, *Cimicifuga heracleifolia* 6 g, *C*. *reticulata* 6g, *Bupleurum chinense* 6 g, and *Angelica sinensis* 3 g	Unclear	Orally	OXA+CAP: OXA: 130 mg/m^2^, d1; CAP: 1,000 mg/m^2^, bid, d1–14, 21 d/C, 2 Cycles	6 w	O1,2,6
Hu et al., 2017	> 60	PT	18/18	22/14	IIIa: 11, IIIb: 14, IV: 11	69.33 ± 9.61/71.60 ± 6.724	Kangai Injection: 50 ml, qd, d1–14, 21 d/C, 6 Cycles/ An approved drug and has a marketing authorization in China	*P*. *ginseng*, *A*. *mongholicus*, *Sophora flavescens*	*Ginsenosides (Rf*, *Rb2*, *Rc*, *Rd*, and *Rb1)* [a]	Intravenously	OXA+S-1: OXA:130 mg/m^2^, d1; S-1: 120 mg/d, d1–14, 21 d/C, 6 Cycles	18 w	O1,2,3
Li et al., 2015	> 60	PT	40/40	49/31	III:56, IV:24	54.6 ± 5.6/55.2 ± 6.4	*Ginseng*-containing Decoction: 100 ml, tid, 28 d/C, 2 Cycles/ Unclear	*P*. *ginseng* 10 g, *O*. *japonicus* 12 g, *Schisandra chinensis* 15 g, *A*. *mongholicus* 30 g, *Dioscorea opposita* 15 g, *C*. *lacryma-jobi var*. *ma-yuen* 30 g, *P*. *cocos* 20 g, *Cornus officinalis* 15 g, *Rehmannia glutinosa* 20 g, *Agrimonia pilosa* 30 g, *Equus asinus* 12 g, *Angelica sinensis* 6 g, *Panax notoginseng* 10 g, *Salvia miltiorrhiza* 15 g, *Paeonia lactiflora*15 g, *P*. *ternate* 7 g, *Bambusa tuldoides* 10 g, *Scutellaria barbata*15 g, *Hedyotis diffusa* 30 g, and *G*. *uralensis* 6 g	Unclear	Orally	TAX+5-Fu+DDP: TAX: 30–50 mg/m^2^, 3 h/d, d1, d8, d15; 5-Fu: 750 mg/m^2^, d1–5; DDP: 20 mg/m^2^, d1–5, 28 d/C, 2 Cycles	8 w	O1,2,3,5
Li et al., 2012	≥ 70	PT	67/68	79/56	IIIb: 39, IIIc: 41, IV: 55	28–79/31–76	Shenfu Injection: 50 ml, qd, d1–7, 21/C, 3 Cycles/ An approved drug and has a marketing authorization in China	*P*. *ginseng* and *Aconitum carmichaelii*	*Ginsenosides (Rg1*, *Re*, *Rb1*, *Rk1*, *20(R)-Rh1*, and *20(S)-Rg3)*, *Panoxatriol*, *(3R*, *9R*, and *10R)-Panaxytriol*, *Panaxydol*, *Heptadeca-1*,*8-dien-4*,*6-diyne-3*,*10-diol*, *Falcarinol* [c]	Intravenously	OXA+CAP: OXA: 100–130 mg/m^2^, d1; CAP: 1,000 mg/m^2^, bid, d1–14, 21d/C, 3 Cycles	9 w	O1,2,3,4
Li, 2020	≥ 60	PT	32/30	41/21	IV	66.74 ± 7.35/67.20 ± 6.92	Aidi Injection: 40 ml, qd, 21 d/C, 4 Cycles/ An approved drug and has a marketing authorization in China	*P*. *ginseng*, *A*. *mongholicus*, *Mylabris*, and *E*. *senticosus*	*Ginsenosides (Rg1*, *Re*, and *Rb1)* [c]	Intravenously	OXA+S-1: OXA:130 mg/m^2^, d1; S-1: body surface area < 1.25 m^2^: 40 mg, bid; 1.25 m^2^ ≤ body surface area: 60 mg, bid; d1–14, 21 d/C, 4 Cycles	12 w	O1,2,3
Liang, 2014	> 70	Unclear	60/60	67/53	IIIb, IV	49.5 ± 7.8/50.2 ± 5.9	Aidi Injection: 50–100 ml, qd, d1–15, 30 d/C, 3 Cycles/ An approved drug and has a marketing authorization in China	*P*. *ginseng*, *A*. *mongholicus*, *Mylabris*, and *E*. *senticosus*	*Ginsenosides (Rg1*, *Re*, and *Rb1)* [c]	Intravenously	S-1: body surface area < 1.25 m^2^: 40 mg, bid; 1.25 m^2^ < body surface area < 1.5 m^2^: 50 mg, bid; 1.5 m^2^ < body surface area: 60 mg, bid; d1–28, 42 d/C, 3 Cycles	18 w	O1,2,3
Lin, 2018	Unclear	Unclear	41/41	45/37	IV	62.0 ± 5.0/62.0 ± 4.5	Fufang Banmao Capsules: 750 mg, tid, 21 d/C, 2 Cycles/ An approved drug and has a marketing authorization in China	*P*. *ginseng*, *A*. *mongholicus*, *Mylabris*, *E*. *senticosus*, *Sparganium stoloniferum*, *Scutellaria barbata*, *Curcuma phaeocaulis*, *Cornus officinalis*, *Ligustrum lucidum*, *BEAR BILE POWDER*, and *G*. *uralensis*	Unclear	Orally	DTX+DDP+5-Fu: DTX: 75 mg/m^2^, d1; DDP: 75 mg/m^2^; 5-Fu: 750 mg/m^2^, d1–5, 21 d/C, 2 Cycles	6 w	O3,5
Liu et al., 2009	≥ 60	PT	30/30	34/26	IIIb: 31, IV: 29	Unclear	Aidi Injection: 50 ml, qd, d1–10, 28 d/C, 2 Cycles/ An approved drug and has a marketing authorization in China	*P*. *ginseng*, *A*. *mongholicus*, *Mylabris*, and *E*. *senticosus*	*Ginsenosides (Rg1*, *Re*, and *Rb1)* [c]	Intravenously	TAX+5-Fu+DDP: TAX:175 mg/m^2^, d1; DDP: 20 mg/m^2^, d1–5; 5-Fu: 600 mg/m^2^, d1–5, 28 d/C, 2 Cycles	8 w	O1,2,3,4
Liu et al., 2019	≥70	PT	60/60	66/54	III:90, IV:30	56.82±7.04/55.23±6.01	Fufang Wannianqing Capsules: 1.2g/d, tid, 21d/C, 3Cycles/ An approved drug and has a marketing authorization in China	*P*. *ginseng*, *Rohdea japonica*, *Scutellaria barbata*, *Curcuma wenyujin*, *Hedyotis diffusa*, *Salvia miltiorrhiza*, *A*. *mongholicus*, *Buthus martensii*, *Polygonum cuspidatum*, and *Scolopendra subspinipes mutilans*	Unclear	Orally	ADM+DDP+Fu: ADM:50 mg/m^2^, d1; DDP: 60 mg/m^2^, d1; Fu: 200 mg/m^2^, 21 d/C, 3 Cycles	9 w	O1,2,4,6
Pei et al., 2013	≥ 60	PT	38/38	43/33	IIIb: 35, IV: 41	65–80/65–79	Yangzheng Xiaoji Capsules: 1.56 g, tid, d1–42, 42 d/C, 2 Cycles/ An approved drug and has a marketing authorization in China	*P*. *ginseng*, *A*. *mongholicus*, *Ligustrum lucidum*, *Curcuma phaeocaulis*, *Ganoderma lucidum*, *Gynostemma pentaphyllum*, *A*. *macrocephala*, *Scutellaria barbata*, *Hedyotis diffusa*, *P*. *cocos*, *Eupolyphaga sinensis Walker*, and *Gallus gallus domesticus Brisson*, *Duchesnea indica*, *Solanum lyratum*, *Artemisia scoparia*, and *Cynanchum paniculatum*	*Ginsenosides (Rg1/Rf*, *Rb1*, *Rb2/Rb3/Rc*, *Ro*, and *F3)*, *Chikusetsusaponin Iva* [48]	Orally	S-1: body surface area < 1.25 m^2^: 40mg, bid; 1.25 m^2^ < body surface area < 1.5 m^2^: 50mg, bid; 1.5 m^2^ < body surface area: 60 mg, bid, d1–28, 42 d/C, 2 Cycles	12 w	O1,2,3
Shi, 2015	Unclear	Unclear	29/29	38/20	III: 27, IV: 31	55.92 ± 6.14/53.28 ± 7.38	Aidi Injection: 50ml, qd, 21d/C, 2Cycles/ An approved drug and has a marketing authorization in China	*P*. *ginseng*, *A*. *mongholicus*, *Mylabris*, *E*. *senticosus*	*Ginsenosides (Rg1*, *Re*, and *Rb1)* [c]	Intravenously	5-Fu+DDP: 5-Fu: 500 mg/m^2^, d1-5; DDP: 50 mg/m^2^, d1–3, 21 d/C, 2 Cycles	6 w	O1,2,5
Tian et al., 2004	> 50	Unclear	23/22	30/15	III:19, IV:16	52.4/53.1	Aidi Injection: 50 ml, qd, d1–40, 40. d/C, 1 Cycles/ An approved drug and has a marketing authorization in China	*P*. *ginseng*, *A*. *mongholicus*, *Mylabris*, and *E*. *senticosus*	*Ginsenosides (Rg1*, *Re*, and *Rb1)* [c]	Intravenously	5-Fu+DDP: 5-Fu: 500 mg/m^2^, d1–5; DDP: 50 mg/m^2^, d1–3, 21 d/C, 2 Cycles	6 w	O1,2,3,4,5
Wang, 2013	≥ 70	PT	32/32	38/26	III, IV	Unclear	Aidi Injection: 60 ml, qd, d1–14, 21 d/C, 2 Cycles/ An approved drug and has a marketing authorization in China	*P*. *ginseng*, *A*. *mongholicus*, *Mylabris*, and *E*. *senticosus*	*Ginsenosides (Rg1*, *Re*, and *Rb1)* [c]	Intravenously	CAP: 1,250 mg/m^2^, bid, d1–14, 21 d/C, 2 Cycles	6 w	O1,2
Wang et al., 2007	≥ 70	PT	32/32	44/20	III, IV	Unclear	Aidi Injection: 60ml, qd, d1–14, 21 d/C, 6 Cycles/ An approved drug and has a marketing authorization in China	*P*. *ginseng*, *A*. *mongholicus*, *Mylabris*, and *E*. *senticosus*	*Ginsenosides (Rg1*, *Re*, and *Rb1)* [c]	Intravenously	OXA+CAP: OXA:120 mg/m^2^, d1; CAP: 1,250 mg/m^2^, bid, d1–14, 21 d/C, 6 Cycles	18 w	O1,2
Wei et al., 2018	Unclear	Unclear	42/42	66/18	IV	Unclear	*Ginseng*-containing Decoction: 200 ml, bid, 14 d/C, 3 Cycles/ Unified production by the hospital	*P*. *ginseng 30 g*, *P*. *cocos 30 g*, *A*. *macrocephala 30 g*, *G*. *uralensis 30 g*, *Angelica sinensis 30 g*, *Rehmannia glutinosa 30 g*, *Ligusticum chuanxiong 30 g*, and *Paeonia lactiflora 30 g*	Unclear	Orally	OXA+CF+5-Fu: OXA: 150 mg/m^2^, 2h/d, d1; CF: 300 mg/m^2^, 2 h/d, d1; 5-Fu: 1,500 mg/m^2^, d1, 14 d/C, 3 Cycles	6 w	O1
Wen et al., 2010	≥ 60	Unclear	27/29	46/10	IV	Unclear	Aidi Injection: 80 ml, qd, d1–21, 28 d/C, 2 Cycles/ An approved drug and has a marketing authorization in China	*P*. *ginseng*, *A*. *mongholicus*, *Mylabris*, and *E*. *senticosus*	*Ginsenosides (Rg1*, *Re*, and *Rb1)* [c]	Intravenously	CF+5-Fu+DDP: CF: 25 mg/(m^2^·d), d1–5/w, 3 w; DDP:10 mg/d, d1–5/w, 3 w; 5-Fu:375 mg/d, d1–21; 28 d/C, 2 Cycles	12 w	O3
Wu and Chen, 2017	> 60	PT	40/40	48/32	IIIa: 30, IIIb: 38, IV: 12	Unclear	*Ginseng*-containing Decoction: 150 ml, bid, 21d/C, 6 Cycles/ Unclear	*P*. *ginseng* 5g, *Curcuma phaeocaulis* 10g, *P*. *cocos* 10 g, *A*. *macrocephala* 10 g, *Portulaca oleracea* 30 g, *C*. *lacryma-jobi var*. *ma-yuen* 30 g, *Hedyotis diffusa* 30 g, *Dolichos lablab* 10 g, *C*. *reticulata* 10 g, *P*. *ternate* 10 g, *Sparganium stoloniferum* 10 g, *Citrus medica* 10 g, *G*. *uralensis* 10 g, *O*. *japonicus* 10 g, *Hordeum vulgare* 15 g, *Setaria italica* 15 g, *Amomum villosum* 3 g	Unclear	Orally	OXA+S-1: OXA: 130 mg/m^2^, 3h/d; S-1: 80 mg/(m^2^·d), d1–14, 21 d/C, 6 ycles	18 w	O1,2,3
Xu, 2015	> 70	Unclear	35/33	32/36	IV	Unclear	*Ginseng* Polydsaochaides Injection: 12 mg/d, d1–14, 14 d/C, 2 Cycles/ An approved drug and has a marketing authorization in China	An Extract of *P*. *ginseng*	*Ginseng Polydsaochaides*	Intravenously	OXA+CF+Fu: OXA:85 mg/m^2^, d1; CF: 300 mg/m^2^, d1–2; Fu: 400 mg/m^2^ ivgtt d1–2 first and 600 mg/m^2^ civ 48 h following, 14 /C, 2 Cycles	4 w	O1,2,3,5
Xu and Lu, 2017	≥ 60	Unclear	47/47	55/39	IIIa: 35, IIIb: 47, IV: 12	53.42 ± 3.96/54.29 ± 4.11	Aidi Injection: 50ml, qd, 21d/C, 4Cycles/ An approved drug and has a marketing authorization in China	*P*. *ginseng*, *A*. *mongholicus*, *Mylabris*, and *E*. *senticosus*	*Ginsenosides (Rg1*, *Re*, and *Rb1)* [c]	Intravenously	OXA+S-1: OXA:130 mg/m^2^, d1; S-1: body surface area < 1.25m^2^: 40 mg, bid; 1.25 m^2^ < body surface area < 1.5 m^2^: 50 mg, bid; 1.5 m^2^ < body surface area: 60 mg, bid, d1–14; 21d/C, 4 Cycles	12 w	O1,2,3,6
Yan et al., 2012	> 70	Unclear	32/34	57/9	III, IV	Unclear	Aidi Injection: 100 ml, qd, d1–10, 14 d/C, 1 Cycles/ An approved drug and has a marketing authorization in China	*P*. *ginseng*, *A*. *mongholicus*, *Mylabris*, and *E*. *senticosus*	*Ginsenosides (Rg1*, *Re*, and *Rb1)* [c]	Intravenously	OXA+CF+Fu: OXA: 95 mg/m^2^, 2 h/d, d1; CF: 200 mg/m^2^, 2 h/d, d1–2; Fu: 400 mg/m^2^ first and 600 mg/m^2^ following, d1–2, 14 d/C, 1 Cycles	2 w	O1,2,4,5
Ye et al., 2017	> 80	PT	40/40	50/30	IIIb: 27, IIIcL33IV: 20	Unclear	Shenmai Injection: 50 ml, qd, d1–14, 28 d/C, 2 Cycles/ An approved drug and has a marketing authorization in China	*P*. *ginseng* and *O*. *japonicus*	Unclear	Intravenously	DTX+DDP+5-Fu: DTX: 75 mg/m^2^, d1; DDP: 75 mg/m^2^, d1; 5-Fu: 1,000 mg/m^2^, d1–5, 28 d/C, 2 Cycles	8 w	O1,2,3,4
Zhu et al., 2019	> 60	PT	30/30	48/12	IV	55.36 ± 6.87/54.57 ± 5.84	Aidi Injection: 40 ml, qd, d1–21, 21 d/C, 4 Cycles/ An approved drug and has a marketing authorization in China	*P*. *ginseng*, *A*. *mongholicus*, *Mylabris*, and *E*. *senticosus*	*Ginsenosides (Rg1*, *Re*, and *Rb1)* [c]	Intravenously	S-1+OXA+DTX: S-1: body surface area < 1.25 m^2^: 40mg, bid; 1.25 m^2^ < body surface area < 1.5 m^2^: 50 mg, bid, d1–14; OXA: 130 mg/m^2^, 2 h/d, d1; DTX: 75 mg/m^2^, 1 h/d, d1, 21 d/C, 4 Cycles	12 w	O1,2,3,6

SD, standard deviation; PT, primary treatment; KPS, Karnofsky Performance Status; TP, therapy procedure; PT, primary treatment; G-TMPs, ginseng-containing traditional medicine preparations; E/C, experimental group (G-TMPs combined with fluoropyrimidine-based chemotherapy regimen)/control group (fluoropyrimidine-based chemotherapy regimen alone); M/F, male/female; O, outcome; O1, objective response rate (ORR); O2, disease control rate (DCR); O3, quality of life (QOL); O4, adverse drug reactions (ADRs); O5, the proportion of peripheral blood lymphocytes; O6, the cancer biomarkers.

Of the different G-TMP interventions, 7 trials [25, 27, 30, 31, 33, 38, 40] used oral G-TMPs, whereas 19 trials [20–24, 26, 28, 29, 32, 34–37, 39, 41–45] used intravenous G-TMPs. The dosage forms of G-TMPs used in the trials included five kinds of injections, three kinds of capsules, and some self-prepared herbal decoctions. Eighteen trials [20, 22–24, 26, 28, 29, 32–37, 39, 41–43, 45] described approved G-TMPs with a clear manufacturer name, production batch number, and marketing authorization in China. Active ingredients from ginseng were also identified in these trials but were unclear in the remaining eight trials [21, 25, 27, 30, 31, 38, 40, 44].

### Evaluation of the methodological bias risk

**Fig 2:**
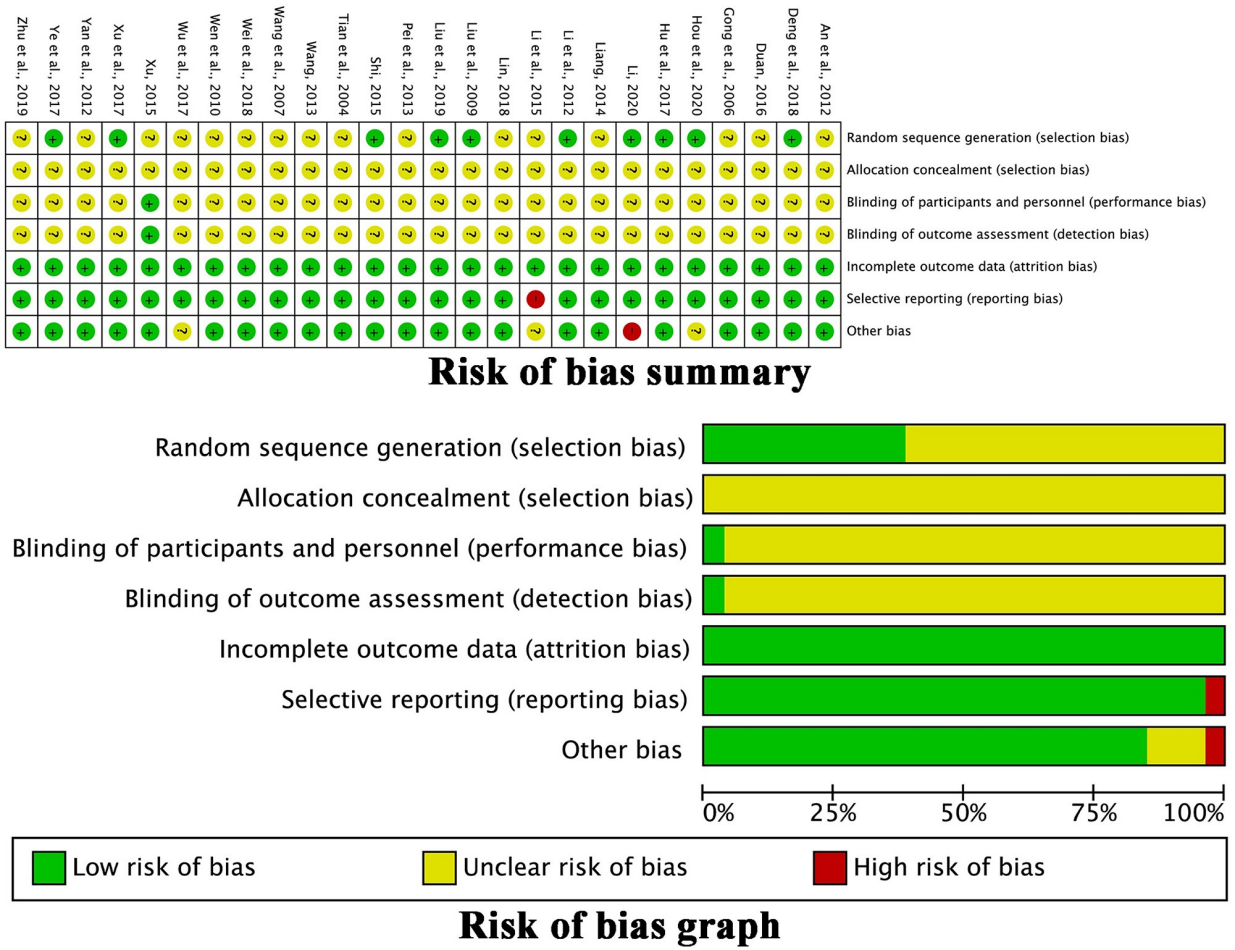
Assessment of methodological bias risk.

Fig 2 depicts the methodological bias risk assessment of each study included. Only nine trials [21, 24–26, 28, 31, 34, 41, 44] reported the random number table or lotteries as methods for random sequence generation. As the other trials did not specify random sequence generation, there was an unknown selection bias. There was no mention of allocation concealment in any of the trials that were included. Except one trial [42], the studies did not report the blinding method, resulting in an ambiguous performance and detection bias. None of the studies reported missing the follow-up. One trial [27] reported incomplete data on the proportions of peripheral blood lymphocytes, and the other trials had a low risk of attrition and reporting bias. Some unclear information, including the duration of intervention, KPS score, therapy procedure, and age, in 19 trials [20, 21, 23, 25, 26, 29, 30, 32, 34–44] might have resulted in other potential biases. Table 1 lists the quality of G-TMPs. Three trials [25, 27, 40] used herbal decoctions, but the origin, processing method, and quality control method were not described. Only one trial [38] described a hospital as a provider of a G-TMP. The other studies focused on a medicine that is authorized in China, including marketing authorization.

### Tumor response

**Fig 3:**
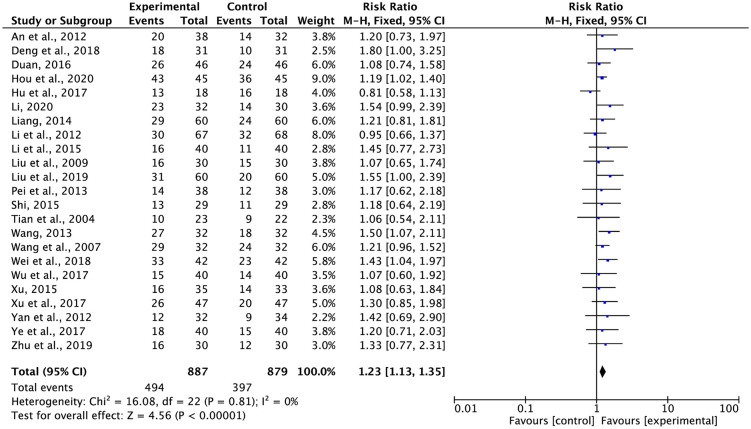
Meta-analysis results of ORR.

**Fig 4:**
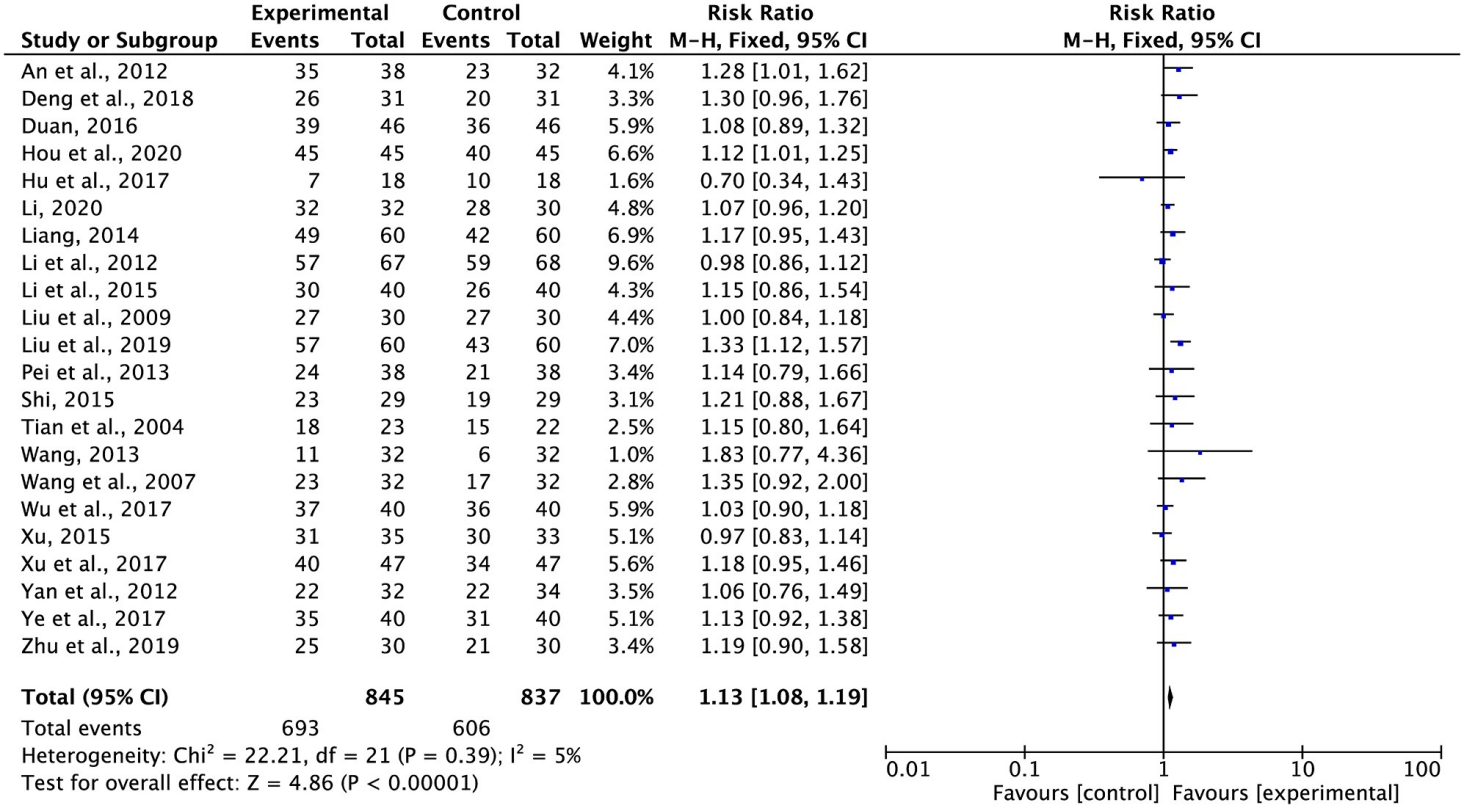
Meta-analysis results of DCR.

ORRs and DCRs were reported in 23 trials with 1,766 and 1,682 patients, respectively, using the WHO and RECIST criteria (Figs 3 and 4). According to Cochran’s Q test and Higgins’ *I*^2^ (*I*^2^ = 0%, *I*^2^ = 5%), there was no heterogeneity across the trials, as illustrated in Figs 3 and 4; hence, data from a variety of tests were combined using the FEM method. The ORR (RR = 1.23, 95% CI: 1.13 to 1.35, p < 0.00001) and DCR (RR = 1.13, 95% CI: 1.08 to 1.19, p < 0.00001) were higher for G-TMPs in combination with FBC than for FBC alone.

### QoL

**Fig 5:**
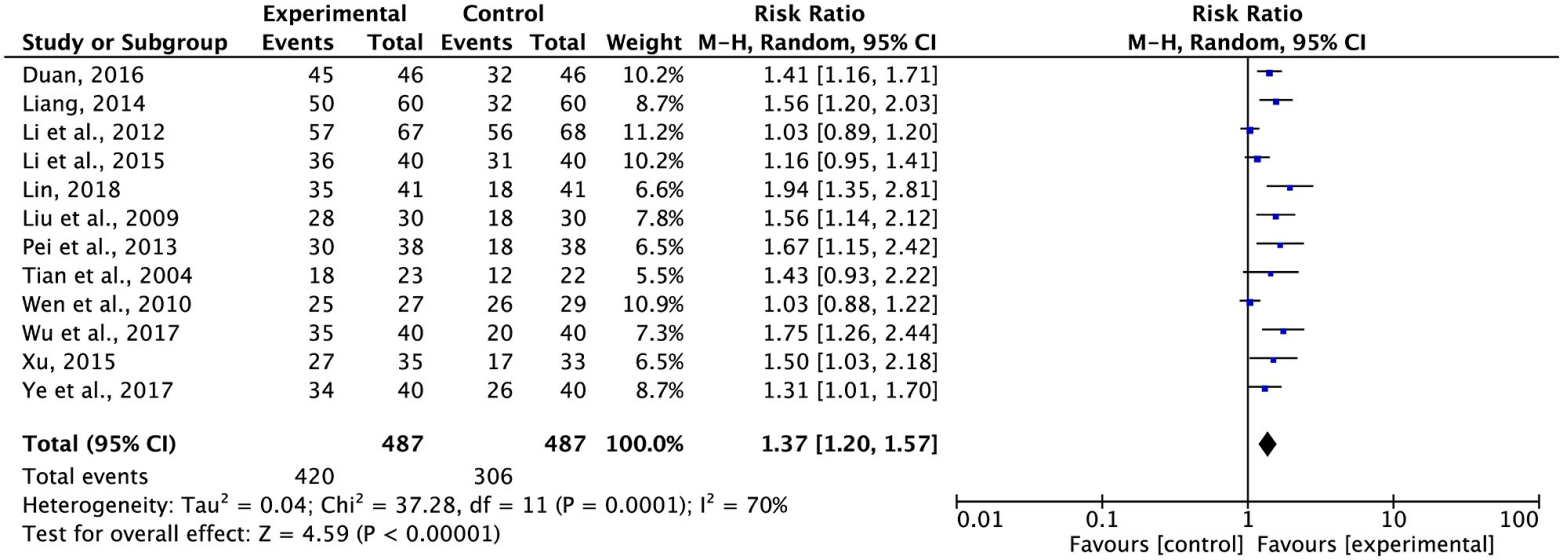
Meta-analysis results of QoL (dichotomous data).

**Fig 6:**
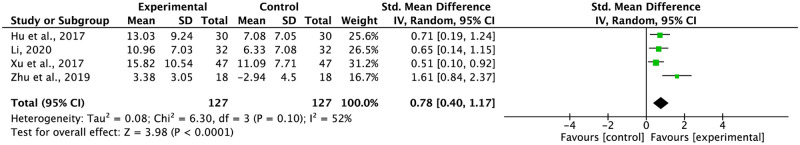
Meta-analysis results of QoL (continuous data).

Based on the KPS scale, 12 studies with 974 participants reported QoL using dichotomous data (Fig 5), while 4 trials with 252 participants reported QoL using continuous data (Fig 6). Owing to a high variability in the dichotomous data (*I*^2^ = 70%), data from separate studies were combined using the REM. Compared with FBC alone, the combination with G-TMPs led to an improvement in the QoL (RR = 1.37, 95% CI: 1.20 to 1.57, p < 0.00001). A subgroup analysis was undertaken to reveal the cause of statistical heterogeneity of these findings (S1 Table). Different types of fluoropyrimidines might have contributed to the disparity in QoL.

A moderate heterogeneity was found in the continuous data for QoL (*I*^2^ = 52%); thus, we used the REM to synthesize data from various studies. The findings revealed that the QoL was improved when G-TMPs were combined with FBC (SMD = 0.78, 95% CI: 0.40 to 1.17, p < 0.0001) compared with that of patients treated with FBC alone. After carefully reading four full articles, we found that three trials [24, 26, 41] used the OXA + S-1 chemotherapy regimen, whereas one trial [45] used the S-1 + OXA + docetaxel chemotherapy regimen. A subgroup study was undertaken to examine whether chemotherapy regimens contributed to the heterogeneity. The *I*^2^ value was reduced from 52 to 0%, indicating that chemotherapy may have been the cause of the QoL heterogeneity.

### Peripheral blood lymphocyte levels

**Fig 7:**
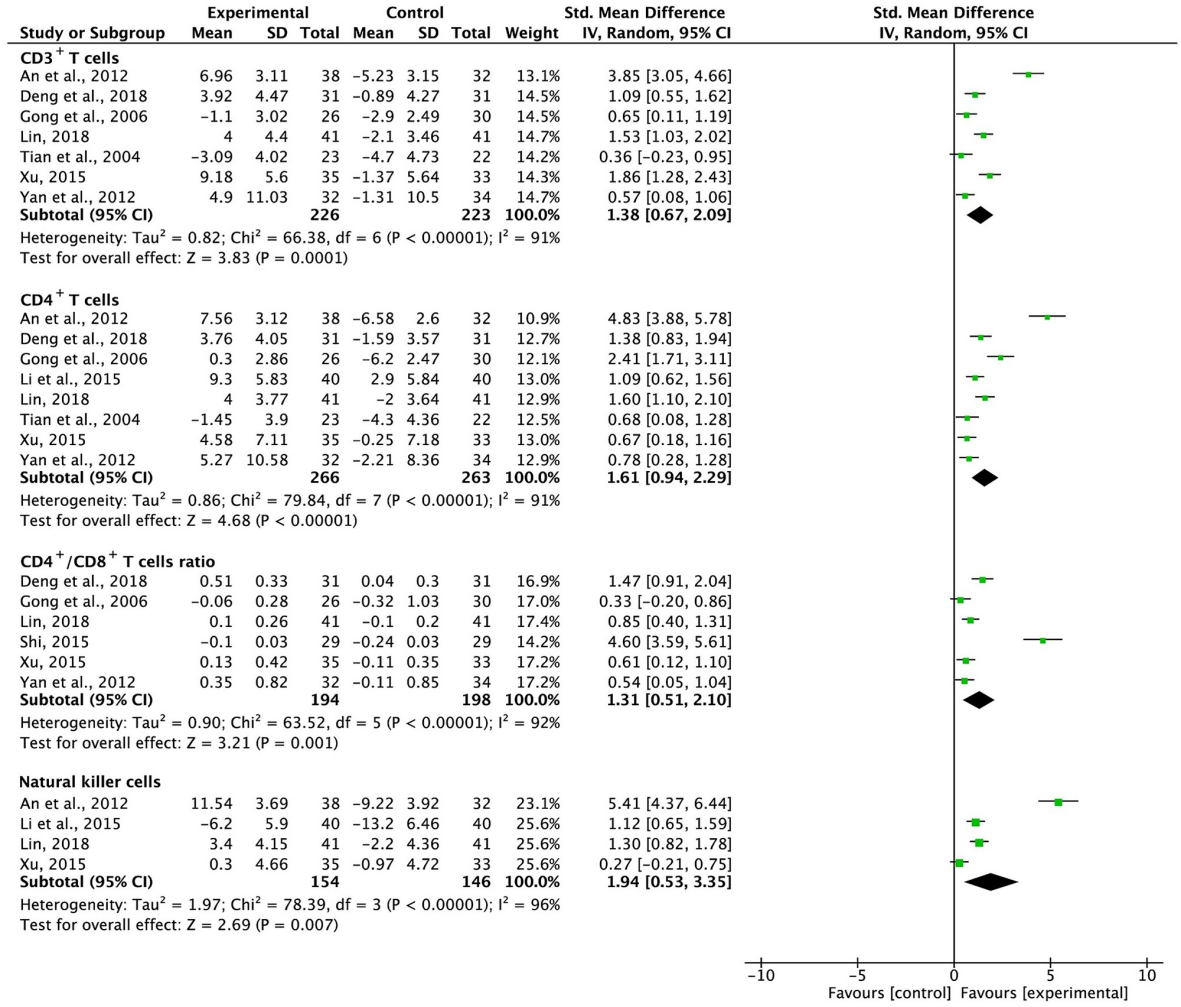
Meta-analysis results of the proportion of peripheral blood lymphocytes.

Nine trials with 587 participants reported the proportions of peripheral blood lymphocytes (Fig 7). Statistical heterogeneity was observed in the CD3^+^ T cell (*I*^2^ = 91%), CD4^+^ T cell (*I*^2^ = 91%), and NK cell (*I*^2^ = 96%) levels and in the CD4^+^/CD8^+^ T-cell ratio (*I*^2^ = 92%); hence, the SMD was synthesized using the REM. The results indicated that the use of G-TMPs in combination with FBC increased the proportions of CD3^+^ T cells (SMD = 1.38, 95% CI: 0.67 to 2.09, p = 0.0001), CD4^+^ T cells (SMD = 1.61, 95% CI: 0.94 to 2.29, p < 0.00001), and NK cells (SMD = 1.94, 95% CI: 0.53 to 3.35, p = 0.007), as well as the CD4^+^/CD8^+^ T-cell ratio (SMD = 1.31, 95% CI: 0.51 to 2.10, p = 0.001), compared with those in the FBC alone group. A subgroup analysis was undertaken to reveal the cause of statistical heterogeneity of these findings (S2 Table). The variability in the proportion of CD3^+^ T cells might have been caused by the therapy regimen. Furthermore, the follow-up time may have caused the heterogeneity in the proportion of CD4^+^ T cells; platinum usage may have caused the heterogeneity in the CD4^+^/CD8^+^ T-cell ratio; the KPS score and follow-up time may have caused the heterogeneity in NK-cell count.

### Cancer biomarkers

**Fig 8:**
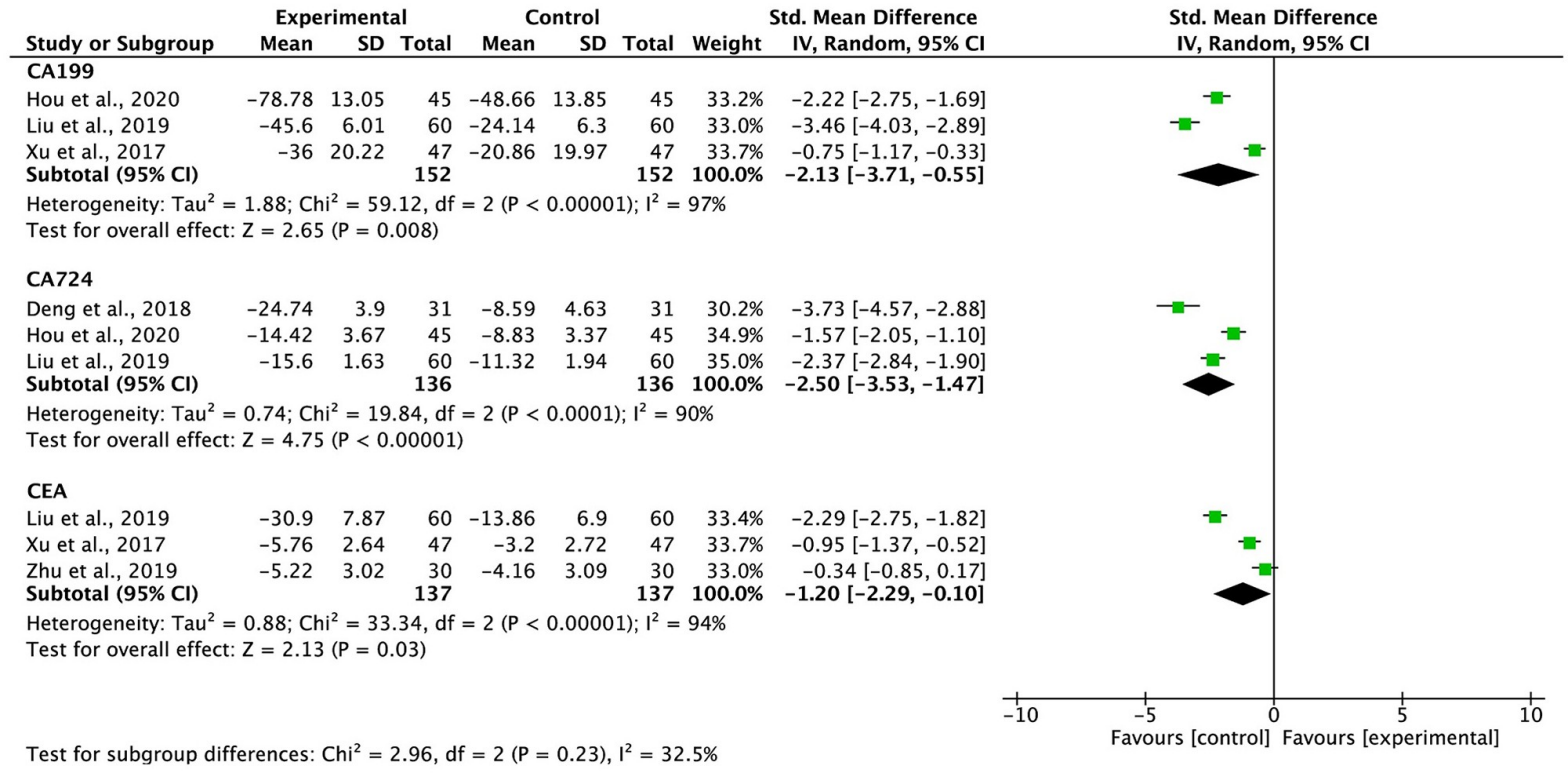
Meta-analysis results of cancer biomarkers.

The biomarkers for cancer were measured in five studies, including a total of 426 participants (Fig 8). The CA19-9 (*I*^2^ = 97%), CEA (*I*^2^ = 94%), and CA72-4 (*I*^2^ = 90%) levels showed statistical heterogeneity; therefore, we used the REM to synthesize the SMD. The findings indicated that compared FBC alone, G-TMPs in combination with FBC reduced the levels of CA19-9 (SMD = -2.13, 95% CI: -3.71 to -0.55, p = 0.008), CA72-4 (SMD = -2.50, 95% CI: -3.53 to -1.47, p < 0.00001), and CEA (SMD = -1.20, 95% CI: -2.29 to -1.10, p = 0.03).

### ADRs

**Fig 9:**
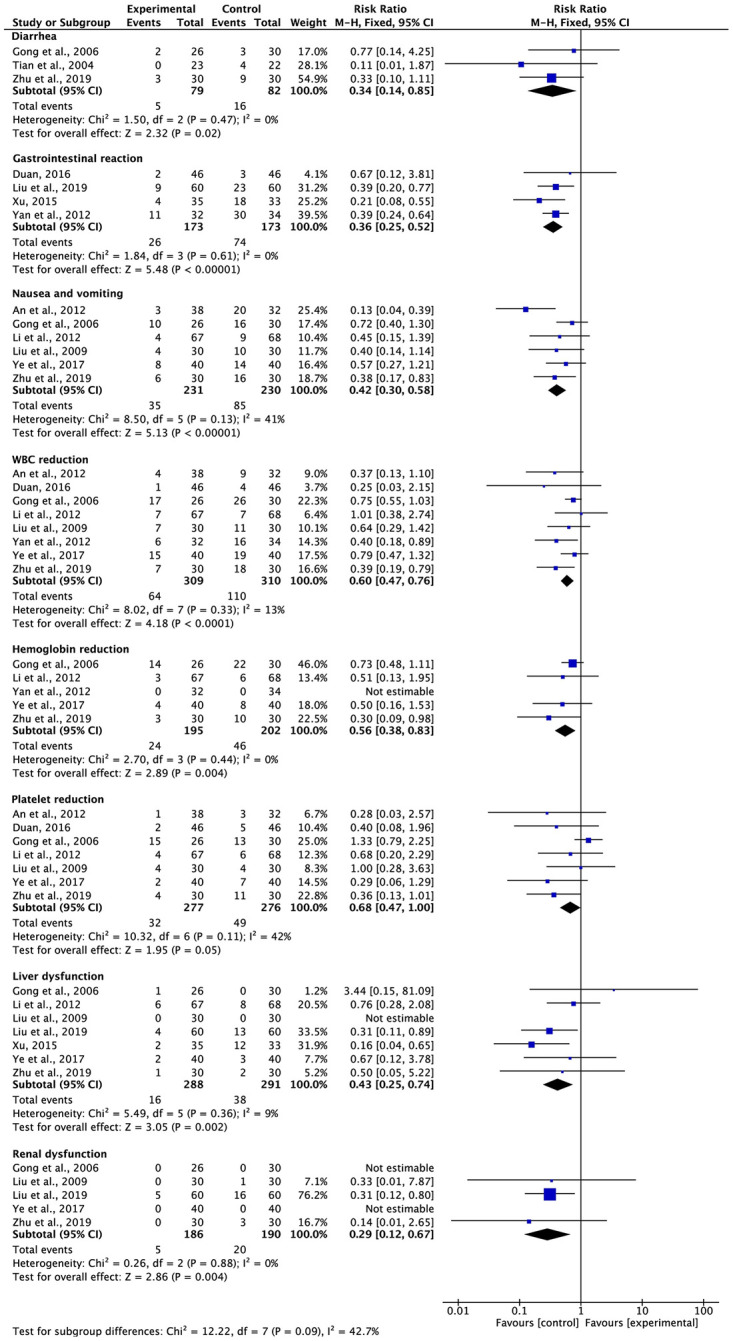
Meta-analysis results of ADRs.

Three studies with 161 participants mentioned diarrhea, four studies with 346 participants mentioned gastrointestinal reactions, six studies with 461 participants reported nausea and vomiting, eight studies with 619 participants reported WBC count reduction, five studies with 397 participants reported hemoglobin level reduction, seven studies with 553 participants reported platelet count reduction, seven studies with 579 participants reported liver dysfunction, and five studies with 376 participants reported renal dysfunction (Table 2 and Fig 9). A moderate heterogeneity was observed in nausea and vomiting (*I*^2^ = 41%) and platelet count reduction (*I*^2^ = 42%), with a minimal heterogeneity for WBC count reduction (*I*^2^ = 13%) and liver dysfunction (*I*^2^ = 9%), while no heterogeneity (*I*^2^ = 0%) was observed in diarrhea, gastrointestinal reactions, hemoglobin reduction, or renal dysfunction. To compile the findings from various studies, the FEM was utilized. The findings indicated that G-TMPs in combination with FBC reduced the risk of diarrhea (RR = 0.34, 95% CI: 0.14 to 0.85, p = 0.02), gastrointestinal reactions (RR = 0.36, 95% CI: 0.25 to 0.52, p < 0.00001), nausea and vomiting (RR = 0.42, 95% CI: 0.30 to 0.58, p < 0.00001), WBC count reduction (RR = 0.60, 95% CI: 0.47 to 0.76, p < 0.0001), hemoglobin reduction (RR = 0.56, 95% CI: 0.38 to 0.83, p = 0.004), platelet count reduction (RR = 0.68, 95% CI: 0.47 to 1.00, p = 0.05), liver dysfunction (RR = 0.43, 95% CI: 0.25 to 0.74, p = 0.002), and renal dysfunction (RR = 0.29, 95% CI: 0.12 to 0.67, p = 0.004) compared with that in patients on FBC alone.

**Table 2:** Meta-analysis results of adverse drug reactions.

Outcomes	Number of trials	Experimental group (Events/Total)	Control group (Events/Total)	SM	RR, 95% CI	Z	*p*	Heterogeneity	
								I^2^	*P*_*h*_
Diarrhea	3	79/161	82/161	FEM	0.34 [0.14, 0.85]	2.32	0.02	0%	0.47
Gastrointestinal reaction	4	173/346	173/346	FEM	0.36 [0.25, 0.52]	5.48	<0.00001	0%	0.61
Nausea and vomiting	6	231/461	230/461	FEM	0.42 [0.30, 0.58]	5.13	<0.00001	41%	0.13
WBC reduction	8	309/619	310/619	FEM	0.60 [0.47, 0.76]	4.18	<0.0001	13%	0.33
Hemoglobin reduction	5	195/397	202/397	FEM	0.56 [0.38, 0.83]	2.89	0.004	0%	0.44
Platelet reduction	7	277/553	276/553	FEM	0.68 [0.47, 1.00]	1.95	0.05	42%	0.11
Liver function disfunction	7	288/579	291/579	FEM	0.43 [0.25, 0.74]	3.05	0.002	9%	0.36
Renal function disfunction	5	186/376	190/376	FEM	0.29 [0.12, 0.67]	2.86	0.004	0%	0.88

RR, risk ratio; CI, confidence interval; SM, statistical method; FEM, fixed-effects model.

### Subgroup analysis

The ORR and DCR were divided into subgroups based on the KPS, therapy procedure, G-TMP drug delivery, fluoropyrimidine usage, platinum usage, and follow-up time (Tables 3 and 4). There were three subgroups based on the KPS score: ≥ 50 or ≥ 60, ≥ 70 or ≥ 80, and unclear. G-TMPs improved the ORR and DCR across all subgroups. Based on the therapy procedures, individuals were classified as primary treatment (PT) and unclear. G-TMPs raised the ORR and DCR in patients, irrespective of whether their therapy procedures were PT or uncertain. Both intravenous and oral administration of G-TMPs raised the ORR and DCR. The use of fluoropyrimidines was divided into three subgroups, namely, 5-FU-, S-1-, and CAP-based chemotherapy regimens. Analysis showed that G-TMPs raised the ORR and DCR in patients, irrespective of the fluoropyrimidine used. There were three subgroups based on the use of platinum, namely, DDP- and OXA-based chemotherapy regimens and no use of platinum. G-TMPs improved the ORR and DCR, irrespective of the platinum use. Three separate follow-up periods were established: ≤ 6 weeks, > 6 and ≤ 12 weeks, and > 12 and ≤ 18 weeks. Only when the follow-up duration was less than 12 weeks did G-TMPs raise the ORR and DCR, according to the subgroup analysis.

**Table 3:** Subgroup analysis of the ORR.

Subgroups	Number of trials	RR (95% CI)	Z	*p*	Heterogeneity	
					I^2^	*P*_*h*_
**Table 3a. Subgroups analysis according to KPS score**						
KPS score (≥50 or ≥60)	11	1.22 [1.05, 1.42]	2.56	0.01	1%	0.43
KPS score (≥70 or ≥80)	9	1.23 [1.08, 1.39]	3.23	0.001	0%	0.78
Unclear	3	1.31 [1.02, 1.68]	2.09	0.04	0%	0.76
**Table 3b. Subgroups analysis according to therapy procedure**						
therapy procedure (PT)	14	1.20 [1.07, 1.35]	3.09	0.002	0%	0.50
Unclear	9	1.28 [1.11, 1.47]	3.46	0.0005	0%	0.91
**Table 3c. Subgroups analysis according to drug delivery of TCM**						
Intravenously	17	1.20 [1.08, 1.34]	3.29	0.001	0%	0.68
Orally	6	1.31 [1.12, 1.53]	3.36	0.0008	0%	0.72
**Table 3d. Subgroups analysis according to the use of fluoropyrimidine**						
Fu-based chemotherapy regimen	11	1.25 [1.08, 1.46]	2.99	0.003	0%	0.97
S-1-based chemotherapy regimen	8	1.25 [1.05, 1.49]	2.57	0.01	25%	0.23
CAP-based chemotherapy regimen	4	1.18 [1.03, 1.35]	2.36	0.02	10%	0.34
**Table 3e. Subgroups analysis according to the use of platinum**						
DDP-based chemotherapy regimen	7	1.23 [1.02, 1.49]	2.14	0.03	0%	0.89
OXA-based chemotherapy regimen	12	1.19 [1.07, 1.33]	3.10	0.002	0%	0.52
None use of platinum	4	1.38 [1.09, 1.73]	2.71	0.007	0%	0.64
**Table 3f. Subgroups analysis according to Follow-up time**						
≤6w	9	1.24 [1.09, 1.41]	3.22	0.001	0%	0.91
6w< and ≤12w	10	1.29 [1.10, 1.50]	3.22	0.001	0%	0.73
12w< and ≤18w	4	1.10 [0.91, 1.33]	1.00	0.32	27%	0.25

RR, risk ratio; CI, confidence interval; ORR, objective response rate; PT, primary treatment.

**Table 4:** Subgroup analysis of the DCR.

Subgroups	Number of trials	RR (95% CI)	Z	*p*	Heterogeneity	
					I^2^	*P*_*h*_
**Table 4a. Subgroups analysis according to KPS score**						
KPS score (≥50 or ≥60)	11	1.10 [1.02, 1.19]	2.57	0.01	0%	0.80
KPS score (≥70 or ≥80)	9	1.14 [1.06, 1.22]	3.63	0.0003	41%	0.09
Unclear	2	1.25 [1.03, 1.52]	2.27	0.02	0%	0.78
**Table 4b. Subgroups analysis according to therapy procedure**						
therapy procedure (PT)	14	1.13 [1.06, 1.20]	3.74	0.0002	24%	0.19
Unclear	8	1.14 [1.05, 1.24]	3.10	0.002	0%	0.68
**Table 4c. Subgroups analysis according to drug delivery of TCM**						
Intravenously	17	1.12 [1.05, 1.19]	3.70	0.0002	0%	0.47
Orally	5	1.16 [1.06, 1.27]	3.32	0.0009	31%	0.21
**Table 4d. Subgroups analysis according to the use of fluoropyrimidine**						
Fu-based chemotherapy regimen	10	1.14 [1.06, 1.23]	3.55	0.0004	15%	0.30
S-1-based chemotherapy regimen	8	1.12 [1.03, 1.22]	2.62	0.009	0%	0.62
CAP-based chemotherapy regimen	4	1.12 [1.01, 1.25]	2.09	0.04	49%	0.12
**Table 4e. Subgroups analysis according to the use of platinum**						
DDP-based chemotherapy regimen	7	1.16 [1.06, 1.26]	3.34	0.0008	0%	0.43
OXA-based chemotherapy regimen	11	1.09 [1.02, 1.16]	2.55	0.01	4%	0.40
None use of platinum	4	1.24 [1.05, 1.45]	2.58	0.01	0%	0.71
**Table 4f. Subgroups analysis according to Follow-up time**						
≤6w	8	1.14 [1.04, 1.24]	2.89	0.004	0%	0.50
6w< and ≤12w	10	1.13 [1.06, 1.21]	3.68	0.0002	24%	0.22
12w< and ≤18w	4	1.10 [0.97, 1.26]	1.50	0.13	24%	0.26

RR, risk ratio; CI, confidence interval; DCR, disease control rate; PT, primary treatment.

As previous described [49, 50], a subgroup analysis was conducted on particular components of ginseng-containing preparations from each trial, as listed in Table 1, to determine the combination of ginseng and other herbs with FBC with the most contribution to AGC treatment. Tables 5 and 6 only include RRs that had a low heterogeneity (*I*^2^ < 30%) and were less than the overall pooled RR. A total of 51 herbs were used, of which more commonly used herbs in combination with ginseng in the treatment of AGC were as follows: *Astragalus mongholicus* Bunge (Fabaceae), *Eleutherococcus senticosus* (Rupr. & Maxim.) Maxim. (Araliaceae), and *Mylabris* (Meloidae). Table 5 shows that when eight herbs were combined with ginseng, they demonstrated significant RRs in benefit for ORR. The most frequently used herb was *A*. *mongholicus* (n = 16; RR 1.25 [1.12, 1.40], *I*^2^ = 0%), while *Citrus reticulata* Blanco (Rutaceae) (n = 2) had the lowest RR (1.16 [0.95, 1.41], *I*^2^ = 0%). These combinations were paired with each other, and nine combinations had significant RRs in benefit for ORR. The most frequently used combinations were as follows: *P*. *ginseng + A*. *mongholicus + E*. *senticosus* (n = 11; RR 1.26 [1.10, 1.43], *I*^2^ = 0%), *P*. *ginseng + A*. *mongholicus + Mylabris* (n = 11; RR 1.26 [1.10, 1.43], *I*^2^ = 0%), and *P*. *ginseng + E*. *senticosus + Mylabris* (n = 11; RR 1.26 [1.10, 1.43], *I*^2^ = 0%). The combinations of *P*. *ginseng + Atractylodes macrocephala* Koidz. (Asteraceae) *+ C*. *reticulata* (n = 2) and *P*. *ginseng + Glycyrrhiza uralensis* Fisch. ex DC. (Fabaceae) *+ C*. *reticulata* (n = 2) had the lowest RRs (1.16 [0.95, 1.41], *I*^2^ = 0%). Furthermore, three combinations of four plants each had significant RRs in benefit for ORR. The most frequently used combination was *P*. *ginseng + A*. *mongholicus + E*. *senticosus + Mylabris* (n = 11; RR 1.26 [1.10, 1.43], *I*^2^ = 0%). The combination of *P*. *ginseng + A*. *macrocephala + G*. *uralensis + C*. *reticulata* (n = 2) had the lowest RR (1.16 [0.95, 1.41], *I*^2^ = 0%). Table 6 shows that 11 herbs exhibited substantial RRs in DCR benefits in combination with ginseng. The most frequently used herb was *A*. *mongholicus* (n = 16; RR 1.17 [1.09, 1.25], *I*^2^ = 0%), while *Citrus reticulata* (n = 2; RR 1.08 [0.99, 1.18], *I*^2^ = 0%), *Coix lacryma-jobi* var. *ma-yuen* (Rom. Caill.) Stapf (Poaceae) (n = 2; RR 1.08 [0.93, 1.25], *I*^2^ = 0%), and *Pinellia ternate* (Thunb.) Breit. (Araceae) (n = 2; RR 1.08 [0.93, 1.25], *I*^2^ = 0%) had the lowest RR. These combinations were paired with each other, and 19 combinations had significant RRs in benefit for DCR. The most frequently used combinations were as follows: *P*. *ginseng + A*. *mongholicus + E*. *senticosus* (n = 11; RR 1.15 [1.06, 1.25], *I*^2^ = 0%), *P*. *ginseng + A*. *mongholicus + Mylabris* (n = 11; RR 1.15 [1.06, 1.25], *I*^2^ = 0%), and *P*. *ginseng + E*. *senticosus + Mylabris* (n = 11; RR 1.15 [1.06, 1.25], *I*^2^ = 0%). The combination of *P*. *ginseng + Poria cocos* (Schw.) Wolf (Polyporaceae) *+ A*. *macrocephala* (n = 2) had the lowest RR (1.07 [0.91, 1.26], *I*^2^ = 0%). Furthermore, 11 combinations of four plants each had significant RRs in benefit for the DCR. The most frequently used combination was *P*. *ginseng + A*. *mongholicus + E*. *senticosus + Mylabris* (n = 11; RR 1.15 [1.06, 1.25], *I*^2^ = 0%). Li et al. [27] and Wu and Chen [40] used the same six G-TMP components, and their herb combination was thus immediately generalized to level 5. The combination was *P*. *ginseng + Ophiopogon japonicus* (L. f.) Ker Gawl. (Liliaceae) *+ G*. *uralensis + P*. *cocos + C*. *lacryma-jobi* var. *ma-yuen + P*. *ternate* (n = 2; RR 1.08 [0.93, 1.25], *I*^2^ = 0%).

**Table 5:** Effects of specific G-TMPs on ORR for AGC: Combination of *Ginseng* and other herbs.

Level	TMPs	RR (95% CI)	N. stud. [Ref]	N. part.	I^2^
1	*P*. *ginseng* + *A*. *mongholicus*	1.25 [1.12, 1.40]	16 [20, 22, 24, 26, 27, 29, 31–37, 41, 43, 45]	1167	0
1	*P*. *ginseng* + *E*. *senticosus*	1.26 [1.10, 1.43]	11 [22, 26, 29, 32, 34–37, 41, 43, 45]	785	0
1	*P*. *ginseng* + *Mylabris*	1.26 [1.10, 1.43]	11 [22, 26, 29, 32, 34–37, 41, 43, 45]	785	0
1	*P*. *ginseng* + *A*. *macrocephala*	1.24 [1.04, 1.46]	4 [25, 33, 38, 40]	330	0
1	*P*. *ginseng* + *G*. *uralensis*	1.27 [1.08, 1.51]	4 [25, 27, 38, 40]	334	0
1	*P*. *ginseng* + *C*. *reticulata*	1.16 [0.95, 1.41]	2 [25, 40]	170	0
1	*P*. *ginseng* + *C*. *lacryma-jobi var*. *ma-yuen*	1.24 [0.81, 1.90]	2 [27, 40]	160	0
1	*P*. *ginseng* + *P*. *ternate*	1.24 [0.81, 1.90]	2 [27, 40]	160	0
2	*P*. *ginseng* + *A*. *mongholicus + E*. *senticosus*	1.26 [1.10, 1.43]	11 [22, 26, 29, 32, 34–37, 41, 43, 45]	785	0
2	*P*. *ginseng* + *A*. *mongholicus* + *Mylabris*	1.26 [1.10, 1.43]	11 [22, 26, 29, 32, 34–37, 41, 43, 45]	785	0
2	*P*. *ginseng* + *E*. *senticosus + Mylabris*	1.26 [1.10, 1.43]	11 [22, 26, 29, 32, 34–37, 41, 43, 45]	785	0
2	*P*. *ginseng* +*A*. *macrocephala + G*. *uralensis*	1.25 [1.05, 1.47]	3 [25, 38, 40]	254	0
2	*P*. *ginseng + A*. *macrocephala + C*. *reticulata*	1.16 [0.95, 1.41]	2 [25, 40]	170	0
2	*P*. *ginseng + G*. *uralensis+ C*. *reticulata*	1.16 [0.95, 1.41]	2 [25, 40]	170	0
2	*P*. *ginseng + G*. *uralensis+ C*. *lacryma-jobi var*. *ma-yuen*	1.24 [0.81, 1.90]	2 [27, 40]	160	0
2	*P*. *ginseng + G*. *uralensis+ P*. *ternate*	1.24 [0.81, 1.90]	2 [27, 40]	160	0
2	*P*. *ginseng + C*. *lacryma-jobi var*. *ma-yuen + P*. *ternate*	1.24 [0.81, 1.90]	2 [27, 40]	160	0
3	*P*. *ginseng + A*. *mongholicus + E*. *senticosus + Mylabris*	1.26 [1.10, 1.43]	11 [22, 26, 29, 32, 34–37, 41, 43, 45]	785	0
3	*P*. *ginseng +A*. *macrocephala + G*. *uralensis + C*. *reticulata*	1.16 [0.95, 1.41]	2 [25, 40]	170	0
3	*P*. *ginseng + C*. *lacryma-jobi var*. *ma-yuen + P*. *ternate + G*. *uralensis*	1.24 [0.81, 1.90]	2 [27, 40]	160	0

TMPs, traditional medicine preparations; RR, risk ratio; CI, confidence interval; N. stud., number of studies; N. part, number of participants; Ref., reference.

**Table 6:** Effects of specific G-TMPs on DCR for AGC: Combination of *Ginseng* and other herbs.

Level	TMPs	RR (95% CI)	N. stud. (Ref)	N. part.	I^2^
1	*P*. *ginseng* + *A*. *mongholicus*	1.17 [1.09, 1.25]	16 [20, 22, 24, 26, 27, 29, 31–37, 41, 43, 45]	1167	0
1	*P*. *ginseng* + *E*. *senticosus*	1.15 [1.06, 1.25]	11 [22, 26, 29, 32, 34–37, 41, 43, 45]	785	0
1	*P*. *ginseng* + *Mylabris*	1.15 [1.06, 1.25]	11 [22, 26, 29, 32, 34–37, 41, 43, 45]	785	0
1	*P*. *ginseng* + *O*. *japonicus*	1.13[1.01, 1.27]	4 [21, 27, 40, 44]	302	0
1	*P*. *ginseng* + *G*. *uralensis*	1.10 [0.99, 1.21]	3 [25, 27, 40]	250	0
1	*P*. *ginseng* + *P*. *cocos*	1.10 [0.95, 1.27]	3 [27, 33, 40]	236	0
1	*P*. *ginseng* + *A*. *macrocephala*	1.09 [0.98, 1.22]	3 [25, 33, 40]	246	0
1	*P*. *ginseng* + *A*. *sinensis*	1.14 [0.99, 1.30]	2 [25, 27]	170	0
1	*P*. *ginseng* + *C*. *reticulata*	1.08 [0.99, 1.18]	2 [25, 40]	170	1
1	*P*. *ginseng* + *C*. *lacryma-jobi var*. *ma-yuen*	1.08 [0.93, 1.25]	2 [27, 40]	160	0
1	*P*. *ginseng* + *P*. *ternate*	1.08 [0.93, 1.25]	2 [27, 40]	160	0
2	*P*. *ginseng* + *A*. *mongholicus* + *E*. *senticosus*	1.15 [1.06, 1.25]	11 [22, 26, 29, 32, 34–37, 41, 43, 45]	785	0
2	*P*. *ginseng* + *A*. *mongholicus* + *Mylabris*	1.15 [1.06, 1.25]	11 [22, 26, 29, 32, 34–37, 41, 43, 45]	785	0
2	*P*. *ginseng* + *E*. *senticosus*+ *Mylabris*	1.15 [1.06, 1.25]	11 [22, 26, 29, 32, 34–37, 41, 43, 45]	785	0
2	*P*. *ginseng* + *A*. *mongholicus + P*. *cocos*	1.15 [0.91, 1.45]	2 [27, 33]	156	0
2	*P*. *ginseng* + *O*. *japonicus + G*. *uralensis*	1.08 [0.93, 1.25]	2 [27, 40]	160	0
2	*P*. *ginseng* + *O*. *japonicus + P*. *cocos*	1.08 [0.93, 1.25]	2 [27, 40]	160	0
2	*P*. *ginseng* + *O*. *japonicus + C*. *lacryma-jobi var*. *ma-yuen*	1.08 [0.93, 1.25]	2 [27, 40]	160	0
2	*P*. *ginseng* + *O*. *japonicus* + *P*. *ternate*	1.08 [0.93, 1.25]	2 [27, 40]	160	0
2	*P*. *ginseng* + *G*. *uralensis* + *P*. *cocos*	1.08 [0.93, 1.25]	2 [27, 40]	160	0
2	*P*. *ginseng* + *G*. *uralensis* + *A*. *macrocephala*	1.08 [0.99, 1.18]	2 [25, 40]	170	1
2	*P*. *ginseng* + *G*. *uralensis* + *A*. *sinensis*	1.14 [0.99, 1.30]	2 [25, 27]	170	0
2	*P*. *ginseng* + *G*. *uralensis* + *C*. *reticulata*	1.08 [0.99, 1.18]	2 [25, 40]	170	1
2	*P*. *ginseng* + *G*. *uralensis* + *C*. *lacryma-jobi var*. *ma-yuen*	1.08 [0.93, 1.25]	2 [27, 40]	160	0
2	*P*. *ginseng* + *G*. *uralensis*+ *P*. *ternate*	1.08 [0.93, 1.25]	2 [27, 40]	160	0
2	*P*. *ginseng* + *P*. *cocos* + *A*. *macrocephala*	1.07 [0.91, 1.26]	2 [33, 40]	156	0
2	*P*. *ginseng* + *P*. *cocos* + *C*. *lacryma-jobi var*. *ma-yuen*	1.08 [0.93, 1.25]	2 [27, 40]	160	0
2	*P*. *ginseng* + *P*. *cocos* + *P*. *ternate*	1.08 [0.93, 1.25]	2 [27, 40]	160	0
2	*P*. *ginseng* + *A*. *macrocephala* + *C*. *reticulata*	1.08 [0.99, 1.18]	2 [25, 40]	170	1
2	*P*. *ginseng* + *C*. *lacryma-jobi var*. *ma-yuen* + *P*. *ternate*	1.08 [0.93, 1.25]	2 [27, 40]	160	0
3	*P*. *ginseng* + *A*. *mongholicus* + *E*. *senticosus* + *Mylabris*	1.15 [1.06, 1.25]	11 [22, 26, 29, 32, 34–37, 41, 43, 45]	785	0
3	*P*. *ginseng* + *O*. *japonicus + G*. *uralensis* + *P*. *cocos*	1.08 [0.93, 1.25]	2 [27, 40]	160	0
3	*P*. *ginseng* + *O*. *japonicus + G*. *uralensis* + *C*. *lacryma-jobi var*. *ma-yuen*	1.08 [0.93, 1.25]	2 [27, 40]	160	0
3	*P*. *ginseng* + *O*. *japonicus + G*. *uralensis* + *P*. *ternate*	1.08 [0.93, 1.25]	2 [27, 40]	160	0
3	*P*. *ginseng* + *O*. *japonicus + P*. *cocos* + *C*. *lacryma-jobi var*. *ma-yuen*	1.08 [0.93, 1.25]	2 [27, 40]	160	0
3	*P*. *ginseng* + *O*. *japonicus + P*. *cocos* + *P*. *ternate*	1.08 [0.93, 1.25]	2 [27, 40]	160	0
3	*P*. *ginseng* + *O*. *japonicus + C*. *lacryma-jobi var*. *ma-yuen* + *P*. *ternate*	1.08 [0.93, 1.25]	2 [27, 40]	160	0
3	*P*. *ginseng* + *G*. *uralensis*+ *P*. *cocos* + *C*. *lacryma-jobi var*. *ma-yuen*	1.08 [0.93, 1.25]	2 [27, 40]	160	0
3	*P*. *ginseng* + *G*. *uralensis*+ *P*. *cocos* + *P*. *ternate*	1.08 [0.93, 1.25]	2 [27, 40]	160	0
3	*P*. *ginseng* + *G*. *uralensis* + *C*. *lacryma-jobi var*. *ma-yuen* + *P*. *ternate*	1.08 [0.93, 1.25]	2 [27, 40]	160	0
3	*P*. *ginseng* + *P*. *cocos* + *C*. *lacryma-jobi var*. *ma-yuen* + *P*. *ternate*	1.08 [0.93, 1.25]	2 [27, 40]	160	0
5	*P*. *ginseng* + *O*. *japonicus* + *G*. *uralensis* + *P*. *cocos* + *C*. *lacryma-jobi var*. *ma-yuen* + *P*. *ternate*	1.08 [0.93, 1.25]	2 [27, 40]	160	0

### Sensitivity analysis

By removing each study, we investigated the robustness of the data by examining the sensitivity of the ORR and DCR, and the results indicated that the pooled RR values for the ORR and DCR were steady.

### Publication bias

**Fig 10:**
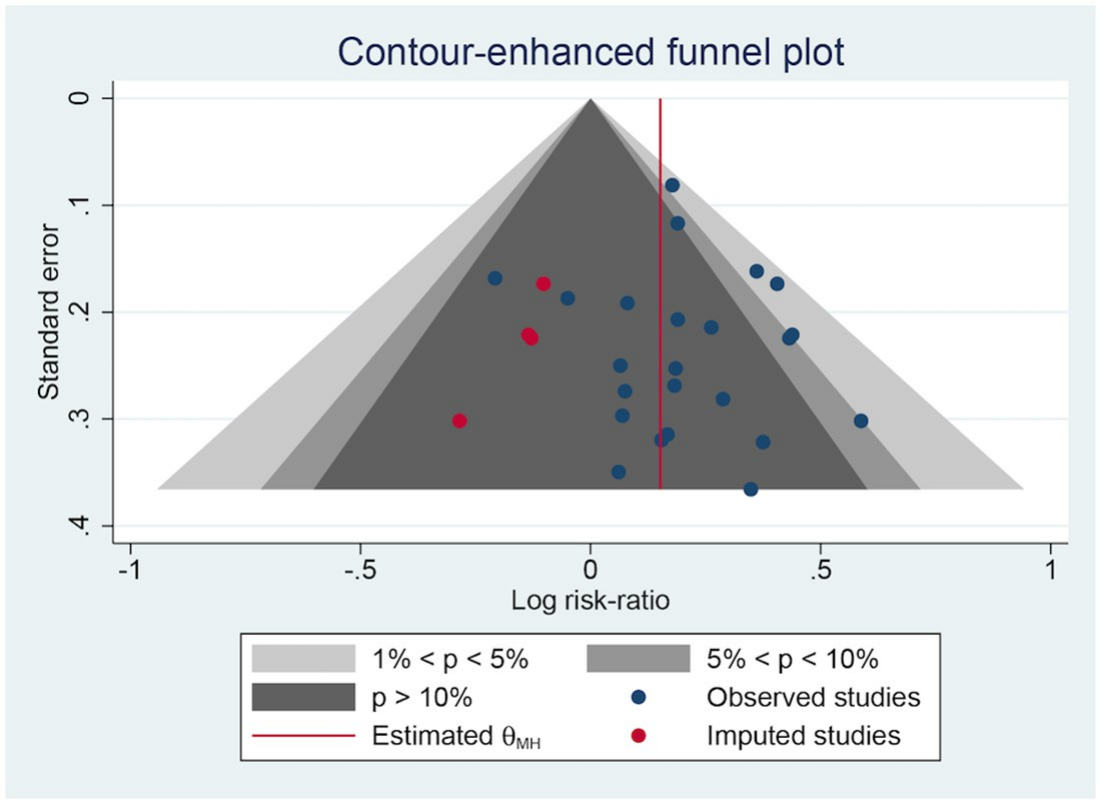
The contour-enhanced plot of the ORR.

**Fig 11:**
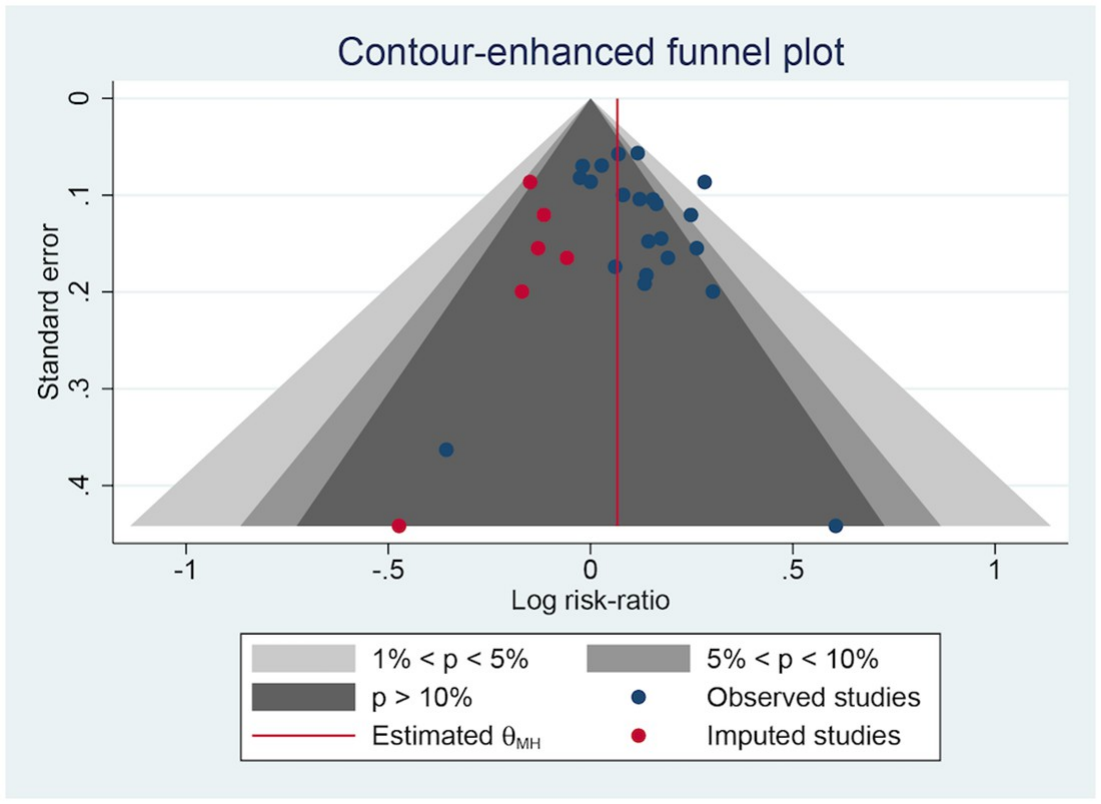
The contour-enhanced plot of the DCR.

**Fig 12:**
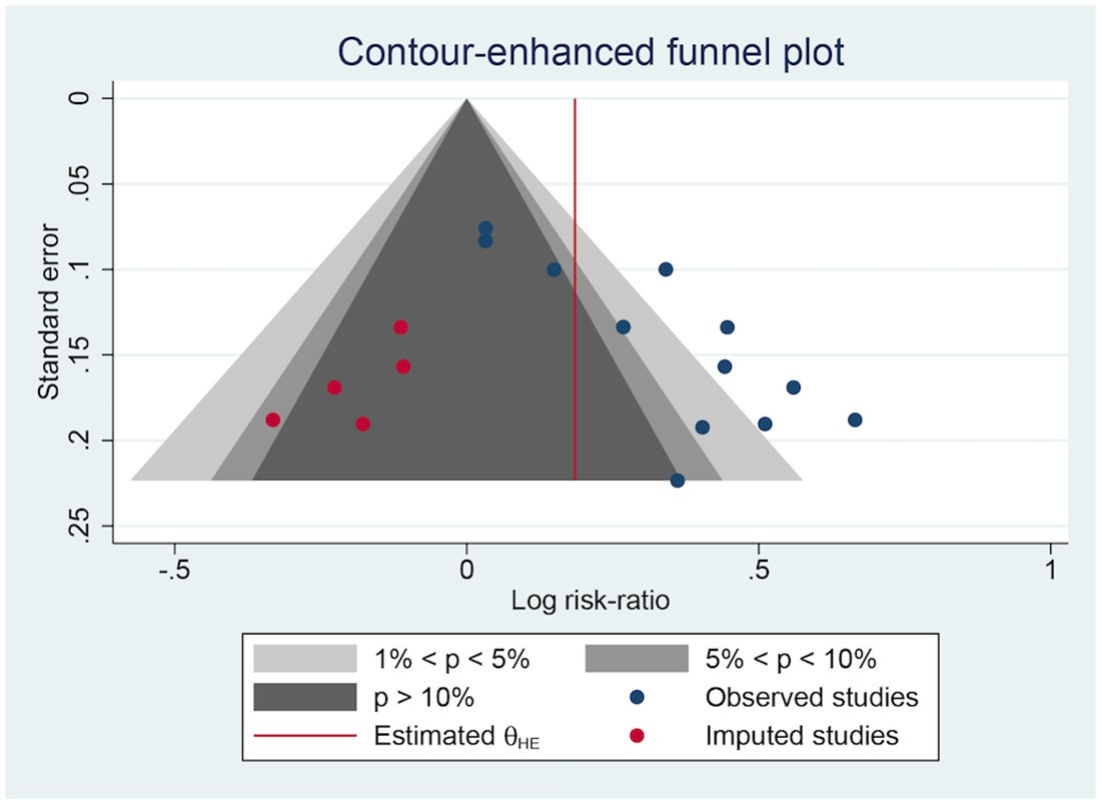
The contour-enhanced plot of the QoL.

Several negative results did not appear to have been published, which might have resulted in publication bias, based on the contour-enhanced plots of the ORR (Fig 10), DCR (Fig 11), and QoL (Fig 12). Egger’s test (Table 7) found no significant publication bias in the ORR (p = 0.6105) and DCR (p = 0.1103) but a strong one in the QoL (p < 0.0001).

**Table 7:** Egger’s test of ORR, DCR and QOL.

Indicators	P value
ORR	0.6105
ORR	0.1103
QOL	0.0000

ORR, objective response rate; DCR, disease control rate; QOL, quality of life

### Quality of evidence

As demonstrated in Tables 8 and 9, the quality of evidence for the ORR, DCR, gastrointestinal reactions, nausea and vomiting, WBC count reduction, hemoglobin reduction, levels of CD3^+^ T, CD4^+^ T, and NK cells, as well as the CD4^+^/CD8^+^ T-cell ratio, was moderate. Meanwhile, the quality of evidence for the QoL (dichotomous and continuous data), platelet count reduction, liver dysfunction, renal dysfunction, and CA72-4 levels was low, while that for diarrhea and CA19-9 and CEA levels was very low.

**Table 8:** GRADE evidence profile of clinical efficacy and safety.

Outcomes (Trials)	Quality assessment					No. of patients		Effect		Quality of evidence
	Risk of bias	Inconsistency	Indirectness	Imprecision	Publication bias	G-TMPs	FBC	Risk ratios (95% CI)	Anticipated absolute effects	
ORR (23)	Serious^a^	NO	NO	NO	NO	494/887 (55.7%)	397/879 (45.2%)	RR 1.23 (1.13–1.35)	104 more per 1,000 (from 59 more to 158 more)	ÅÅÅO MODERATE
DCR (22)	Serious^a^	NO	NO	NO	NO	693/845 (82%)	606/837 (72.4%)	RR 1.13 (1.08–1.19)	94 more per 1,000 (from 58 more to 138 more)	ÅÅÅO MODERATE
QOL (dichotomous data) (12)	Serious^a^	NO^c^	NO	NO	Serious^d^	420/487 (86.2%)	306/487 (62.8%)	RR 1.37 (1.20–1.57)	232 more per 1,000 (from 126 more to 358 more)	ÅÅOO LOW
Diarrhea (3)	very serious^b^	NO	NO	Serious^e^	NO	5/79 (6.3%)	16/82 (19.5%)	RR 0.34 (0.14, 0.85)	129 fewer per 1,000 (from 29 fewer to 168 fewer)	ÅOOO VERY LOW
Gastrointestinal reaction (4)	Serious^a^	NO	NO	NO	NO	26/173 (15%)	74/173 (42.8%)	RR 0.36 (0.25, 0.52)	274 fewer per 1,000 (from 205 fewer to 321 fewer)	ÅÅÅO MODERATE
Nausea and vomiting (6)	Serious^a^	NO	NO	NO	NO	35/231 (15.2%)	85/230 (37%)	RR 0.42 (0.30, 0.58)	214 fewer per 1,000 (from 155 fewer to 259 fewer)	ÅÅÅO MODERATE
WBC reduction (8)	Serious^a^	NO	NO	NO	NO	64/309 (20.7%)	110/310 (35.5%)	RR 0.60 (0.47, 0.76)	142 fewer per 1,000 (from 85 fewer to 188 fewer)	ÅÅÅO MODERATE
Hemoglobin reduction (5)	Serious^a^	NO	NO	NO	NO	24/195 (12.3%)	46/202 (22.8%)	RR 0.56 (0.38, 0.83)	100 fewer per 1,000 (from 39 fewer to 141 fewer)	ÅÅÅO MODERATE
Platelet reduction (7)	very serious^b^	NO	NO	NO	NO	32/277 (11.6%)	49/276 (17.8%)	RR 0.68 (0.47, 1.00)	57 fewer per 1,000 (from 94 fewer to 0 more)	ÅÅOO LOW
Liver function disfunction (7)	very serious^b^	NO	NO	NO	NO	16/288 (5.6%)	38/291 (13.1%)	RR 0.43 (0.25, 0.74)	74 fewer per 1,000 (from 34 fewer to 98 fewer)	ÅÅOO LOW
Renal function disfunction (5)	very serious^b^	NO	NO	NO	NO	5/186 (2.7%)	20/190 (10.5%)	RR 0.29 (0.12, 0.67)	75fewer per 1,000 (from 35 fewer to 93 fewer)	ÅÅOO LOW

^a^ Most trials had unclear risk, and with high risk, but the result had good robustness. The evidence was rated down by only one level.

^b^ Most trials had unclear risk, and with high risk, and the result had poor robustness. The evidence was rated down by two level.

^c^ Heterogeneity presented in them, and the results had good robustness. Not rated down.

^d^ There was publication bias. The QOL was over-estimated. The evidence was rated down by one level.

^e^ The sample size for each outcome was fewer than 300 cases. Therefore, the evidence was rated down by one level.

**Table 9:** GRADE evidence profile of QOL (continuous data), peripheral blood lymphocyte levels and cancer biomarkers.

Outcomes (Trials)	Quality assessment					No. of patients		Mean difference (95% CI)	Quality of evidence
	Risk of bias	Inconsistency	Indirectness	Imprecision	Publication bias	G-TMPs	FBC		
QOL (continuous data) (4)	serious^a^	NO^c^	NO	Serious^e^	NO	127	127	SMD 0.78 higher (0.4–1.17 higher)	ÅÅOO LOW
CD3+ T cells (7)	serious^a^	NO^c^	NO	NO	NO	226	223	SMD 1.38 higher (0.67–2.09 higher)	ÅÅÅO MODERATE
CD4+ T cells (8)	serious^a^	NO^c^	NO	NO	NO	266	263	SMD 1.61 higher (0.94–.29 higher)	ÅÅÅO MODERATE
CD4+/CD8+ T cells ratio (6)	serious^a^	NO^c^	NO	NO	NO	194	198	SMD 1.31 higher (0.51–2.1 higher)	ÅÅÅO MODERATE
NK cells (4)	serious^a^	NO^c^	NO	NO	NO	154	146	SMD 1.94 higher (0.53–3.35 higher)	ÅÅÅO MODERATE
CA199 (3)	very serious^b^	serious^d^	NO	NO	NO	152	152	SMD 2.13 lower (3.71–0.55 lower)	ÅOOO VERY LOW
CA724 (3)	serious^a^	NO^c^	NO	Serious^e^	NO	136	136	SMD 2.5 lower (3.53–1.47 lower)	ÅÅOO LOW
CEA (3)	very serious^b^	serious^d^	NO	Serious^e^	NO	137	137	SMD 1.20 lower (2.29–0.10 lower)	ÅOOO VERY LOW

^a^ Most trials had unclear risk, and with high risk, but the result had good robustness. The evidence was rated down by only one level.

^b^ Most trials had unclear risk, and with high risk, and the result had poor robustness. The evidence was rated down by two level.

^c^ Heterogeneity presented in them, and the results had good robustness. Not rated down.

^d^ Heterogeneity presented in them, and the results had poor robustness. The evidence was rated down by one level.

^e^ The sample size for each outcome was fewer than 300 cases. Therefore, the evidence was rated down by one level.

## Discussion

G-TMPs are a complementary and alternative therapy commonly used in patients with cancer to increase the efficacy and decrease the side effects in combination with chemotherapy [51]. In recent years, meta-analyses have confirmed the clear clinical effects of G-TMPs on non-small cell lung cancer [11] and liver cancer [52]. Gastric cancer is a prevalent malignant tumor of the digestive system and is often diagnosed at a late stage, when treatment is typically ineffective. The combination of G-TMPs and FBC has been frequently used to prolong the survival of patients with AGC, although the efficacy of this treatment regimen has not been systematically evaluated. Therefore, we conducted a meta-analysis to determine whether the combination of G-TMPs and FBC is effective in the treatment of AGC. To the best of our knowledge, this is the first thorough review and meta-analysis of RCTs that tested the efficacy of G-TMPs in the treatment of AGC. This meta-analysis evaluated the tumor response, QoL, immune function, cancer biomarkers, and ADRs based on the data from 26 RCTs comprising 1,960 individuals with AGC.

G-TMPs in combination with FBC greatly improved the tumor response, according to our findings. Previous studies have shown a remarkable anti-gastric cancer activity of components of ginseng, which induced tumor cell apoptosis via reactive oxygen species, promoted endoplasmic reticulum stress, and inhibited tumor cell migration and invasion [53–55]. In particular, when ingredients of ginseng are used in combination with 5-FU, they can significantly enhance the inhibition of colorectal [56, 57], liver [58, 59], pancreatic [60], and gastric [61] cancers. These synergistic anticancer effects imply that the combination of G-TMPs with FBC may have therapeutic use for the treatment of cancer. Recent clinical investigations have also supported this conclusion [28, 32, 42]. Based on experimental and clinical evidence, we believe that G-TMPs in combination with FBC might significantly improve the tumor response in patients with AGC.

Combinations of ginseng and other herbs are commonly used in the therapeutic treatment of AGC. Subgroup analyses revealed that in combination with ginseng, the following six herbs exhibited significant combined RRs and no heterogeneity at numerous combination levels: *A*. *mongholicus*, *E*. *senticosus*, *Mylabris*, *A*. *macrocephala*, *G*. *uralensis*, and *O*. *japonicus*. Therefore, combinations of these herbs could be considered particularly effective in improving the tumor response in AGC when combined with chemotherapy. Song et al. [62] concluded that *Astragalus* polysaccharide, a compound extracted from *A*. *mongholicus*, might act as a chemotherapeutic sensitizer by independently inducing the apoptosis of and the proapoptotic effect of doxorubicin on gastric cancer cells. Another study showed that cantharidin, a bioactive component derived from *Mylabris phalerata* Pallas, inhibited the invasion and metastasis of gastric cancer by inhibiting the activation of the PI3K/AKT signaling pathway [63]. Xie et al. [64] found that a combination of liquiritin, a major constituent of *G*. *uralensis*, and a tumor necrosis factor-related apoptosis-inducing ligand acted synergistically to induce the apoptosis of gastric cancer cells by activating caspases. Moreover, atractylenolide II, a major sesquiterpene lactone isolated from *A*. *macrocephala*, inhibited the proliferation and motility and induced the apoptosis of gastric cancer cells, which might be related to the inhibition of the RAS/ERK and PI3K/AKT signaling pathways [65]. Data on the other two herbs as well as on combinations of the six herbs in the treatment of AGC are limited.

Immunological monitoring is of great significance for inhibiting the occurrence and development of tumors and an effective way for assessing immune function by monitoring lymphocyte subtypes in peripheral blood. High levels of T and NK cells are associated with favorable overall and progression-free survival [66], while mice with combined immunodeficiencies in both T and NK cells have increased susceptibility to cancer development [67]. Our results showed that G-TMPs in combination with FBC increased the levels of T and NK cells compared with those in the FBC alone group, indicating the efficacy of G-TMPs in improving immune function and prognosis of patients with AGC.

Serum tumor markers play a significant role in detecting recurrence and distant metastasis and in predicting the survival of patients with gastrointestinal malignant tumors [68]. Monitoring the combination of CA19-9, CA72-4, and CEA is recommended for tumor staging of gastric cancer before surgery or chemotherapy and can be used to detect cancer recurrence or assess its response [69]. Our results showed that G-TMPs in combination with FBC significantly reduced the levels of CA19-9, CA72-4, and CEA compared with those in the FBC alone group, indicating a positive effect of G-TMPs on the prognosis of AGC.

ADRs during therapy, including gastrointestinal reactions, myelosuppression, and liver and renal dysfunction, seriously affect patient’s compliance and QoL; hence, this problem must be addressed in clinical research. Our findings suggested that the treatment with G-TMPs reduces the occurrence of diarrhea, gastrointestinal responses, and myelosuppression in patients, as well as their risk of liver and renal failure. Previous experimental studies demonstrated that, *P*. *ginseng* may effectively ameliorate chemotherapy-induced diarrhea by modulating gut microbial structure, when paired with *A*. *macrocephala* [70]. *P*. *ginseng* might also considerably ameliorate chemotherapy-induced myelosuppression by increasing the numbers of the bone marrow nucleated cells and peripheral blood cells, when paired with *O*. *japonicus* [71]. Furthermore, *P*. *ginseng* might attenuate chemotherapy-induced liver injuries by inducing cytochrome P450 expression and mediating the l-arginine/nitric oxide pathway [72], and chemotherapy-induced renal injuries by reducing oxidative stress and preserving antioxidant enzymes [73]. Based on the chemotherapy, adding G-TMPs as an adjuvant therapy not only reduced the occurrence of ADRs but also reduced the incidence of chemotherapy-related side effects.

G-TMP-related adverse effects were not reported in any of the included studies, while some clinical trials have confirmed the safety of ginseng [74, 75]. However, one study reported adverse reactions of a ginseng medicine, which positively correlated with the dosage used and were mainly associated with abnormalities in the thyroid, adrenal, and nervous systems and with oxidative stress [76]. G-TMPs are unlikely to cause these adverse events, but their safety must be evaluated before use. Moreover, since ginseng may induce the activity of CYP3A in the liver and gastrointestinal tract [77], interactions between ginseng and chemotherapy drugs pose a potential risk [78]. Although one study has reported that ginseng might cause hepatotoxicity when combined with imatinib, there are currently no studies reporting G-TMP-caused adverse reactions in combination with FBC. Nevertheless, Clinicians must consider the interactions and adverse effects when delivering G-TMPs in conjunction with FBC.

In addition to ginseng, *A*. *mongholicus*, *Codonopsis pilosula* (Franch.) Nannf. (Campanulaceae), and *G*. *uralensis* have the potential to treat AGC in combination with chemotherapy. Cheng et al. [79] systematically evaluated the combination of *A*. *mongholicus* with platinum-based chemotherapy and showed that it might have a greater efficacy and less adverse effects in the treatment of AGC than chemotherapy alone. Li et al. [80] have previously evaluated combinations of traditional Chinese medicines with paclitaxel-based chemotherapy and listed three significant combinations of herbs, including *C*. *pilosula* and *G*. *uralensis*. Elemenes and cinobufotalin were also shown to have therapeutic affect against AGC, despite not being as tonic as herbs, however, the specific chemotherapeutic drugs to be used in combination with these compounds remain to be determined [81, 82].

Our study has potential limitations. First, since we only considered research published in either English or Chinese language, it is possible that studies in other languages, such as Japanese or Korean, may have been overlooked. Second, only nine studies [21, 24–26, 28, 31, 34, 41, 44] reported random sequence generation, and only one study [42] reported the blinding method, which may have introduced potential selection, performance, and detection biases. As a result, certain outcomes in the final GRADE review were rated as being of poor or very poor quality. Nonetheless, the sensitivity analysis showed that the outcomes were robust and reliable. Our findings will be constantly updated as new high-quality studies are published. Third, there were three outcomes with more than 10 included studies. There was considerable publication bias in the QoL based on the findings of the contour-enhanced plot and Egger’s test, which suggested that some unfavorable outcomes might have not been published. Fourth, nine studies [32, 36–40, 42–44] did not specify the age ranges of the included individuals, which made it impossible to carry out a subgroup analysis to explore the influence of age on the outcomes. Fifth, few studies reported the effect of G-TMPs in combination with FBC on long-term survival of patients with AGC; thus, further research is needed to address this issue. Sixth, the active ingredients of several G-TMPs, including Shenmai Injection, Fufang Banmao Capsules, Fufang Wannianqing Capsules, and all decoctions, remain unclear and require clarification from the drug manufacturers or a research study. In addition, none of the included studies fully recorded the sources, processing methods, and dosages of all herbs in the formulations, and authentication, quality control procedures, and safety monitoring data were not described for self-prepared herbal decoctions, which may have led to a bias.

Future studies must consider the potential value of this treatment against AGC, and follow the CONSORT guidelines [83] for designing and reporting clinical trials to improve the quality of evidence.

## Conclusion

G-TMPs in combination with FBC have the potential to enhance efficacy, reduce ADRs, and improve prognosis for AGC patients. Combinations of these herbs, including *A*. *mongholicus*, *E*. *senticosus*, *Mylabris*, *A*. *macrocephala*, *G*. *uralensis*, and O. *japonicus*, could potentially improve the tumor response to treatment in AGC when combined with chemotherapy. The potential way of the G-TMPs in combination with FBC for reducing ADRs might be related to modulate gut microbial structure for ameliorating diarrhea, increasing the numbers of the bone marrow nucleated cells and peripheral blood cells for ameliorating myelosuppression, induce cytochrome P450 expression and mediate the l-arginine/nitric oxide pathway for ameliorating liver injuries, and reduce oxidative stress and preserve antioxidant enzymes for ameliorating renal injuries. The benefit of G-TMPs paired with FBC for prognosis of AGC might be related to synergistic anticancer effects and improved immune function. Furthermore, the long-term efficacy of G-TMPs in combination with FBC in AGC treatment must be verified via well-designed clinical trials that adhere to CONSORT guidelines in future.

## Supporting information

S1 Table Subgroup analysis of the QoL using dichotomous data. The subgroup analysis of the QoL is available in S1 Table.
(DOCX)

S2 Table Subgroup analysis of the peripheral blood lymphocyte levels. The subgroup analysis of peripheral blood lymphocyte levels is available in S2 Table.
(DOCX)

S1 File Detailed search strategy. The detailed search strategy is available in S1 File.
(DOCX)

S2 File PRISMA checklist. The PRISMA checklist is available in S2 File.
(DOCX)

## Abbreviations

ADRs: Adverse drug reactions
AGC: Advanced gastric cancer
CI: Confidence interval
DCR: Disease control rate
FBC: Fluoropyrimidine-based chemotherapy
FEM: Fixed-effect model
G-TMPs: *Ginseng*-containing traditional medicine preparations
KPS: Karnofsky performance status
NK cells: Natural killer cells
ORR: Objective response rate
PT: Primary treatment
QoL: Quality of life
RCTs: Randomized controlled trials
REM: Random-effect model
RR: Risk ratio
SD: Standard deviation
SM: Statistical method
SMD: Standardized mean difference
TP: therapy procedure
