# Supplementary material for: Ginseng-containing traditional medicine preparations in combination with fluoropyrimidine-based chemotherapy for advanced gastric cancer: A systematic review and meta-analysis
Source: PLoS One. 2023 Apr 17;18(4):e0284398. doi: 10.1371/journal.pone.0284398 (PMC10109524; doi:10.1371/journal.pone.0284398)
Supplement: S1 Table — The subgroup analysis of the QoL is available in S1 Table. (DOCX) [file pone.0284398.s001.docx]

**Table S1. Subgroup analysis of the QoL using dichotomous data**

| **Subgroups** | **Number of trials** | **RR (95% CI)** | **Z** | ***p*** | **Heterogeneity** | | **TSD** |
| --- | --- | --- | --- | --- | --- | --- | --- |
|  |  |  |  |  | **I²** | ***P_h_*** |  |
| **Table S1a. Subgroups analysis according to KPS score** | | | | | | |  |
| KPS score (≥50 or ≥60) | 7 | 1.36 [1.14, 1.63] | 3.47 | 0.0005 | 69% | 0.004 | 42.7% |
| KPS score (≥70 or ≥80) | 4 | 1.30 [1.03, 1.65] | 2.19 | 0.03 | 72% | 0.01 |  |
| Unclear | 1 | 1.94 [1.35, 2.81] | 3.54 | 0.0004 | Not applicable | Not applicable |  |
| **Table S1b. Subgroups analysis according to therapy procedure** | | | | | | |  |
| Treatment process (PT) | 7 | 1.34 [1.15, 1.57] | 3.67 | 0.0002 | 67% | 0.006 | 0% |
| Unclear | 5 | 1.44 [1.06, 1.95] | 2.36 | 0.02 | 81% | 0.0004 |  |
| **Table S1c. Subgroups analysis according to drug delivery of G-TCM** | | | | | | |  |
| Intravenously | 8 | 1.30 [1.12, 1.51] | 3.43 | 0.0006 | 67% | 0.003 | 19.7% |
| Orally | 4 | 1.56 [1.17, 2.09] | 3.01 | 0.003 | 72% | 0.01 |  |
| **Table S1d. Subgroups analysis according to the usage of fluoropyrimidine** | | | | | | |  |
| Fu-based chemotherapy regimen | 8 | 1.35 [1.16, 1.57] | 3.81 | 0.0001 | 64% | 0.008 | 87.2% |
| S-1-based chemotherapy regimen | 3 | 1.64 [1.37, 1.96] | 5.38 | <0.00001 | 0% | 0.87 |  |
| CAP-based chemotherapy regimen | 1 | 1.03 [0.89, 1.20] | 0.43 | 0.67 | Not applicable | Not applicable |  |
| **Table S1e. Subgroups analysis according to the usage of platinum** | | | | | | | |
| DDP-based chemotherapy regimen | 6 | 1.33 [1.09, 1.63] | 2.77 | 0.006 | 72% | 0.004 | 0% |
| OXA-based chemotherapy regimen | 3 | 1.36 [0.92, 2.02] | 1.54 | 0.12 | 83% | 0.003 |  |
| None use of platinum | 2 | 1.60 [1.29, 1.98] | 4.27 | <0.0001 | 0% | 0.78 |  |
| **Table S1f. Subgroups analysis according to follow-up time** | | | | | | |  |
| ≤6w | 4 | 1.50 [1.29, 1.74] | 5.37 | <0.00001 | 0% | 0.46 | 55.8% |
| 6w< and ≤12w | 6 | 1.23 [1.05, 1.45] | 2.51 | 0.01 | 70% | 0.005 |  |
| 12w< and ≤18w | 2 | 1.60 [1.29, 1.98] | 4.27 | <0.0001 | 0% | 0.78 |  |

**Note:** RR: risk ratio, CI: confidence interval, QOL: quality of life, PT: primary treatment, TSD: Test for subgroup differences.
