# Supplementary material for: Ginseng-containing traditional medicine preparations in combination with fluoropyrimidine-based chemotherapy for advanced gastric cancer: A systematic review and meta-analysis
Source: PLoS One. 2023 Apr 17;18(4):e0284398. doi: 10.1371/journal.pone.0284398 (PMC10109524; doi:10.1371/journal.pone.0284398)
Supplement: S2 Table — The subgroup analysis of peripheral blood lymphocyte levels is available in S2 Table. (DOCX) [file pone.0284398.s002.docx]

| **Table S2. Subgroup analysis of the peripheral blood lymphocyte levels** | | | | | | | | |
| --- | --- | --- | --- | --- | --- | --- | --- | --- |
| **Subgroups** | **CD3^+^ T cells** | | **CD4^+^ T cells** | | **CD4+/ CD8+ T cells ratio** | | **NK cell activity** | |
|  | **SMD (95% CI)** | **TSD** | **SMD (95% CI)** | **TSD** | **SMD (95% CI)** | **TSD** | **SMD (95% CI)** | **TSD** |
| **Table S2a. Subgroups analysis according to KPS score** | | | | | | | | |
| KPS score (≥50 or ≥60) | 0.71 [0.30, 1.12] | I^2^=36.5% | 1.36 [0.73, 2.00] | I^2^=60.5% | 0.90 [-0.22, 2.02] | I^2^=0% | 1.12 [0.65, 1.59] | I^2^=74.4% |
| KPS score (≥70) | 1.20 [-0.06, 2.46] |  | 0.72 [0.37, 1.07] |  | 0.58 [0.23, 0.92] |  | 0.27 [-0.21, 0.75] |  |
| Unclear | 2.67 [0.39, 4.95] |  | 3.19 [0.03, 6.35] |  | 2.70 [-0.97, 6.38] |  | 3.33 [-0.69, 7.35] |  |
| **Table S2b. Subgroups analysis according to therapy procedure** | | | | | | | | |
| Treatment process (PT) | 3.85 [3.05, 4.66] | I^2^=97.2% | 2.94 [-0.73, 6.60] | I^2^=0% | NO | Not applicable | 3.24 [-0.96, 7.44] | I^2^=19.2% |
| Unclear | 1.01 [0.55, 1.47] |  | 1.23 [0.73, 1.73] |  | 1.31 [0.51, 2.10] |  | 0.79 [-0.23, 1.80] |  |
| **Table S2c. Subgroups analysis according to drug delivery of G-TCM** | | | | | | | | |
| Intravenously | 1.36 [0.52, 2.21] | I^2^=0% | 1.74 [0.77, 2.70] | I^2^=0% | 1.43 [0.41, 2.45] | I^2^=1.9% | 2.82 [-2.22, 7.85] | I^2^=0% |
| Orally | 1.53 [1.03, 2.02] |  | 1.34 [0.83, 1.85] |  | 0.85 [0.40, 1.31] |  | 1.21 [0.87, 1.55] |  |
| **Table S2d. Subgroups analysis according to the usage of fluoropyrimidine** | | | | | | | | |
| Fu-based chemotherapy regimen | 1.44 [0.60, 2.28] | I^2^=0% | 1.66 [0.88, 2.44] | I^2^=0% | 1.29 [0.35, 2.23] | I^2^=0% | 1.94 [0.53, 3.35] | Not applicable |
| S-1-based chemotherapy regimen | 1.09 [0.55, 1.62] |  | 1.38 [0.83, 1.94] |  | 1.47 [0.91, 2.04] |  | NO |  |
| **Table S2e. Subgroups analysis according to the usage of platinum** | | | | | | | | |
| DDP-based chemotherapy regimen | 0.86 [0.15, 1.56] | I^2^=0% | 1.42 [0.78, 2.06] | I^2^=0% | 1.86 [0.01, 3.72] | I^2^=75.5% | 1.21 [0.87, 1.55] | I^2^=0% |
| OXA-based chemotherapy regimen | 2.07 [0.35, 3.78] |  | 2.04 [0.08, 4.01] |  | 0.58 [0.23, 0.92] |  | 2.82 [-2.22, 7.85] |  |
| None use of platinum | 1.09 [0.55, 1.62] |  | 1.38 [0.83,1.94] |  | 1.47 [0.91, 2.04] |  | NO |  |
| **Table S2e. Subgroups analysis according to follow-up time** | | | | | | | | |
| >4w | 1.46 [0.51, 2.42] | I^2^=0% | 1.94 [1.07, 2.81] | I^2^=84.5% | 1.74 [0.45, 3.02] | I^2^=65.8% | 2.54 [0.69, 4.38] | I^2^=81.6% |
| ≤4w | 1.20 [-0.60, 2.46] |  | 0.72 [0.37, 1.07] |  | 0.58 [0.23, 0.92] |  | 0.27 [-0.21, 0.75] |  |
| **Note:** PT: primary treatment, SMD: standardized mean difference, TSD: Test for subgroup differences | | | | | | | | |
