# Supplementary material for: Ginseng-containing traditional medicine preparations in combination with fluoropyrimidine-based chemotherapy for advanced gastric cancer: A systematic review and meta-analysis
Source: PLoS One. 2023 Apr 17;18(4):e0284398. doi: 10.1371/journal.pone.0284398 (PMC10109524; doi:10.1371/journal.pone.0284398)
Supplement: S1 File — The detailed search strategy is available in S1 File. (DOCX) [file pone.0284398.s003.docx]

**Supplementary material 1. Detailed search strategy**

**Supplementary Table A: Search Strategy Used in PubMed 2021/6/29**

| No. | Search items | Items found |
| --- | --- | --- |
| #1 | "stomach neoplasms"[MeSH Terms] OR (("gastr*"[Title/Abstract] OR "stomach*"[Title/Abstract] OR "digest*"[Title/Abstract] OR "epigastr*"[Title/Abstract]) AND ("carcin*"[Title/Abstract] OR "cancer*"[Title/Abstract] OR "neoplas*"[Title/Abstract] OR "tumour*"[Title/Abstract] OR "tumor*"[Title/Abstract] OR "growth*"[Title/Abstract] OR "adenocarcin*"[Title/Abstract] OR "malig*"[Title/Abstract])) | 279367 |
| #2 | "panax"[MeSH Terms] OR "ginsenosides"[MeSH Terms] OR "renshen"[Title/Abstract] OR "schinseng"[Title/Abstract] OR "panax"[Title/Abstract] OR "ginsan"[Title/Abstract] OR "ginseng*"[Title/Abstract] OR "shinseng"[Title/Abstract] OR "ninjin"[Title/Abstract] OR "gingilone"[Title/Abstract] OR "panaxoside*"[Title/Abstract] OR "ginsenoside*"[Title/Abstract] OR "protopanaxa*"[Title/Abstract] OR "protopanaxadiol"[Title/Abstract] OR "protopanaxatriol"[Title/Abstract] OR "panaxagin"[Title/Abstract] OR "ginsenol"[Title/Abstract] OR "ginsenine"[Title/Abstract] OR "bazhen"[Title/Abstract] OR "shiquandabu"[Title/Abstract] OR "xiaochaihu"[Title/Abstract] OR "biejiajian"[Title/Abstract] OR "buzhongyiqi"[Title/Abstract] OR "guipi"[Title/Abstract] OR "banmao"[Title/Abstract] OR "tianxian"[Title/Abstract] OR "ruanjian"[Title/Abstract] OR "shenyi"[Title/Abstract] OR "fufangteng"[Title/Abstract] OR "qixuekang"[Title/Abstract] OR "shenqi pills"[Title/Abstract] OR "zhenyuan"[Title/Abstract] OR "aidi"[Title/Abstract] OR "delisheng"[Title/Abstract] OR "kangai"[Title/Abstract] OR "yangzheng"[Title/Abstract] OR "hechan"[Title/Abstract] | 13190 |
| #3 | ("randomized controlled trial"[Publication Type] OR "controlled clinical trial"[Publication Type] OR "randomized"[Title/Abstract] OR "placebo"[Title/Abstract] OR "drug therapy"[MeSH Terms] OR "randomly"[Title/Abstract] OR "trial"[Title/Abstract] OR "groups"[Title/Abstract]) NOT ("animals"[MeSH Terms] NOT "humans"[MeSH Terms]) | 3671334 |
| #4 | #1 and #2 and #3 | 71 |

**Supplementary Table B: Search Strategy Used in EMBASE 2021/6/29**

| No. | Search items | Items found |
| --- | --- | --- |
| #1 | 'stomach tumor'/exp | 173,091 |
| #2 | gastr*:ab,ti OR stomach*:ab,ti OR digest*:ab,ti OR epigastr*:ab,ti | 1,213,403 |
| #3 | carcin*:ab,ti OR cancer*:ab,ti OR neoplas*:ab,ti OR tumour*:ab,ti OR tumor*:ab,ti OR growth*:ab,ti OR adenocarcin*:ab,ti OR malig*:ab,ti | 6,110,902 |
| #4 | #2 AND #3 | 376,390 |
| #5 | #1 OR #4 | 414,398 |
| #6 | 'panax'/exp | 11,018 |
| #7 | 'ginsenoside'/exp | 4,432 |
| #8 | panax:ab,ti OR ginsenosides:ab,ti OR renshen:ab,ti OR schinseng:ab,ti OR panax:ab,ti OR ginsan:ab,ti OR ginseng*:ab,ti OR shinseng:ab,ti OR ninjin:ab,ti OR gingilone:ab,ti OR panaxoside*:ab,ti OR ginsenoside*:ab,ti OR protopanaxa*:ab,ti OR protopanaxadiol:ab,ti OR protopanaxatriol:ab,ti OR panaxagin:ab,ti OR ginsenol:ab,ti OR ginsenine:ab,ti OR bazhen:ab,ti OR shiquandabu:ab,ti OR xiaochaihu:ab,ti OR biejiajian:ab,ti OR buzhongyiqi:ab,ti OR guipi:ab,ti OR banmao:ab,ti OR tianxian:ab,ti OR ruanjian:ab,ti OR shenyi:ab,ti OR fufangteng:ab,ti OR qixuekang:ab,ti OR “shenqi pills”:ab,ti OR zhenyuan:ab,ti OR aidi:ab,ti OR delisheng:ab,ti OR kangai:ab,ti OR yangzheng:ab,ti OR hechan:ab,ti | 15,892 |
| #9 | #6 OR #7 OR #8 | 18,930 |
| #10 | random* | 1,910,205 |
| #11 | #5 AND #9 AND #10 | 81 |

**Supplementary Table C: Search Strategy Used in CoChrane 2021/6/29**

| No. | Search items | Items found |
| --- | --- | --- |
| #1 | MeSH descriptor: [Stomach Neoplasms] explode all trees | 2662 |
| #2 | (gastr* OR stomach* OR digest* OR epigastr*):ti,ab,kw | 92272 |
| #3 | (carcin* OR cancer* OR neoplas* OR tumour* OR tumor* OR growth* OR adenocarcin* OR malig*):ti,ab,kw | 260979 |
| #4 | #2 AND #3 | 24793 |
| #5 | #1 OR #4 | 24793 |
| #6 | MeSH descriptor: [Panax] explode all trees | 248 |
| #7 | MeSH descriptor: [Ginsenosides] explode all trees | 55 |
| #8 | ("renshen" OR "schinseng" OR "panax" OR "ginsan" OR "ginseng*" OR "shinseng" OR "ninjin" OR "gingilone" OR "panaxoside*" OR "ginsenoside*" OR "protopanaxa*" OR "protopanaxadiol" OR "protopanaxatriol" OR "panaxagin" OR "ginsenol" OR "ginsenine" OR "bazhen" OR "shiquandabu" OR "xiaochaihu" OR "biejiajian" OR "buzhongyiqi" OR "guipi" OR "banmao" OR "tianxian" OR "ruanjian" OR "shenyi" OR "fufangteng"OR "qixuekang" OR "shenqi pills" OR "zhenyuan" OR "aidi" OR "delisheng" OR "kangai" OR "yangzheng" OR "hechan"):ti,ab,kw | 0 |
| #9 | #6 OR #7 OR #8 | 274 |
| #10 | #5 AND #9 | 5 |
